# Genetic Control of Kinetochore-Driven Microtubule Growth in *Drosophila* Mitosis

**DOI:** 10.3390/cells11142127

**Published:** 2022-07-06

**Authors:** Julia V. Popova, Gera A. Pavlova, Alyona V. Razuvaeva, Lyubov A. Yarinich, Evgeniya N. Andreyeva, Alina F. Anders, Yuliya A. Galimova, Fioranna Renda, Maria Patrizia Somma, Alexey V. Pindyurin, Maurizio Gatti

**Affiliations:** 1 Institute of Molecular and Cellular Biology, Siberian Branch of Russian Academy of Sciences, 630090 Novosibirsk, Russia; popova@mcb.nsc.ru (J.V.P.); gpavlova@ed.ac.uk (G.A.P.); alena.razuvaeva@mcb.nsc.ru (A.V.R.); l.yarinich@mcb.nsc.ru (L.A.Y.); andreeva@mcb.nsc.ru (E.N.A.); alina.munzarova@gmail.com (A.F.A.); galimova@mcb.nsc.ru (Y.A.G.); 2 Laboratory of Bioengineering, Novosibirsk State Agrarian University, 630039 Novosibirsk, Russia; 3 Wellcome Centre for Cell Biology, School of Biological Sciences, University of Edinburgh, Edinburgh EH9 3BF, UK; 4 Institute of Cytology and Genetics, Siberian Branch of Russian Academy of Sciences, 630090 Novosibirsk, Russia; 5 Faculty of Natural Sciences, Novosibirsk State University, 630090 Novosibirsk, Russia; 6 Institute of Molecular Biology and Pathology (IBPM), National Research Council (CNR), c/o Department of Biology and Biotechnology, Sapienza University of Rome, 00185 Rome, Italy; fioranna.renda@gmail.com (F.R.); mariapatrizia.somma@cnr.it (M.P.S.)

**Keywords:** mitosis, S2 cells, *Drosophila*, microtubule regrowth, microtubule depolymerization, colcemid, kinetochores, Mast/Orbit/Chb, Mei-38, Mars, Dgt6, Eb1, Patronin, Asp, Klp10A

## Abstract

Centrosome-containing cells assemble their spindles exploiting three main classes of microtubules (MTs): MTs nucleated by the centrosomes, MTs generated near the chromosomes/kinetochores, and MTs nucleated within the spindle by the augmin-dependent pathway. Mammalian and *Drosophila* cells lacking the centrosomes generate MTs at kinetochores and eventually form functional bipolar spindles. However, the mechanisms underlying kinetochore-driven MT formation are poorly understood. One of the ways to elucidate these mechanisms is the analysis of spindle reassembly following MT depolymerization. Here, we used an RNA interference (RNAi)-based reverse genetics approach to dissect the process of kinetochore-driven MT regrowth (KDMTR) after colcemid-induced MT depolymerization. This MT depolymerization procedure allows a clear assessment of KDMTR, as colcemid disrupts centrosome-driven MT regrowth but not KDMTR. We examined KDMTR in normal *Drosophila* S2 cells and in S2 cells subjected to RNAi against conserved genes involved in mitotic spindle assembly: *mast*/*orbit*/*chb* (*CLASP1*), *mei-38* (*TPX2*), *mars* (*HURP*), *dgt6* (*HAUS6*), *Eb1* (*MAPRE1/EB1*), *Patronin* (*CAMSAP2*), *asp* (*ASPM*), and *Klp10A* (*KIF2A*). RNAi-mediated depletion of Mast/Orbit, Mei-38, Mars, Dgt6, and Eb1 caused a significant delay in KDMTR, while loss of Patronin had a milder negative effect on this process. In contrast, Asp or Klp10A deficiency increased the rate of KDMTR. These results coupled with the analysis of GFP-tagged proteins (Mast/Orbit, Mei-38, Mars, Eb1, Patronin, and Asp) localization during KDMTR suggested a model for kinetochore-dependent spindle reassembly. We propose that kinetochores capture the plus ends of MTs nucleated in their vicinity and that these MTs elongate at kinetochores through the action of Mast/Orbit. The Asp protein binds the MT minus ends since the beginning of KDMTR, preventing excessive and disorganized MT regrowth. Mei-38, Mars, Dgt6, Eb1, and Patronin positively regulate polymerization, bundling, and stabilization of regrowing MTs until a bipolar spindle is reformed.

## 1. Introduction

The spindle is a microtubule (MT)-based highly dynamic molecular machine that mediates precise chromosome segregation during both mitosis and meiosis. To form a spindle, centrosome-containing cells generate MTs in three main cellular locations: at the centrosomes, near chromosomes and/or at kinetochores, and within the spindle through the augmin-mediated pathway (reviewed in [1,2,3,4,5]). MTs are always nucleated by the γ-tubulin ring complexes (γ-TuRCs), which are embedded in the centrosomes, enriched in the vicinity of the kinetochores, or associated with the walls of the spindle MTs by interaction with augmin (reviewed in [3,6]). Studies carried out in mammalian tissue culture cells and in different types of *Drosophila* somatic cells have shown that chromosome/kinetochore-driven MT formation is sufficient for the assembly of a functional spindle, but to date, little is known about the factors that govern the growth of kinetochore-dependent MTs (reviewed in [3,4,6,7,8]).

Early studies using *Xenopus* oocyte extracts revealed that chromatin has the ability to drive MT growth and bipolar spindle formation (reviewed in [9]). In addition, mammalian cells are able to form bipolar spindles after centrosome ablation with laser microsurgery [10]. Consistent with these results, *Drosophila* mutants devoid of centrosomes, or with centrosomes with strongly reduced MT nucleating activity (e.g., *asl* (*CEP152*), *Sas-4* (*CENPJ*), *cnn* (*CDK5RAP2*), and *spd-2* (*CEP192*) mutants; unless mentioned otherwise, here and henceforth, the human ortholog of the fly gene or protein is reported within brackets), can assemble functional mitotic spindles and develop to adulthood [7,11,12,13,14,15]. Centrosomal MTs are also dispensable for spindle formation in *Drosophila* tissue culture cells. For example, S2 cells subjected to RNAi-mediated depletion of centrosomal components, such as Cnn, Sas-4, and Spd-2, assemble functional anastral spindles [16,17,18]. Thus, centrosomes and astral MTs appear to be dispensable for the assembly of a functional spindle in both mammalian and *Drosophila* somatic cells.

Three main approaches are currently used to analyze chromatin/kinetochore-driven MT formation. A first approach involves direct examination of kinetochore fibers (k-fibers) formation from unattached kinetochores in live centrosome-containing cells expressing GFP-tubulin (reviewed in [1]). A second approach exploits systems devoid of functional centrosomes such as *Xenopus laevis* extracts or cells deficient of critical centrosomal proteins required for MT nucleation. A third approach consists in the analysis of spindle MTs regrowth after cold- or drug-induced MT depolymerization. In *Xenopus laevis* extracts, chromatin- or DNA-coated beads stimulate MT nucleation and polymerization along their entire surface (reviewed in [9]). Similarly, in *Drosophila* embryos, MT regrowth occurs throughout mitotic chromatin [19]. In contrast, in S2 cells and human cells, MT growth is restricted to the kinetochore regions [20,21,22,23,24,25,26] (and this study).

Studies on mitosis in *Xenopus* extracts and vertebrate cells have shown that the GTP-bound form of Ran GTPase (RanGTP) stimulates chromatin-induced MT growth. RanGTP is generated in the vicinity of chromosomes by RCC1, a chromosome-associated RanGTP exchange factor (reviewed in [9]). In both *Xenopus* and mammalian systems, RanGTP forms a gradient highly concentrated around the chromosomes that positively regulates several MT-associated proteins including Aurora A, TPX2, HURP, Aurora B, INCENP, and Nup107-160 (reviewed in [27]). The role of RanGTP in chromosome-driven MT growth has been also studied in *Drosophila* embryos and different types of fly somatic cells. Although S2 tissue culture cells form a RanGTP gradient around the chromosomes, RNAi-mediated depletion of >95% of RCC1 does not affect spindle assembly and functioning, and, consistently, it does not result in defective kinetochore-driven MT growth [28]. However, *Drosophila* embryos injected with a dominant negative form of Ran are severely defective in chromosome-driven MT regrowth after cold-induced depolymerization [19,29]. Thus, it is currently unclear whether chromosome-associated MT polymerization in S2 cells requires a minimal concentration of RanGTP, or whether it is RanGTP-independent.

The current model on the role of kinetochores in spindle assembly is largely based on the analysis of mitosis in centrosome-containing *Drosophila* S2 cells expressing GFP-tagged tubulin. Careful observations on mitosis in these live cells, accompanied by laser microsurgery experiments, suggested that the plus ends of short chromatin-induced MTs are captured by the kinetochores and continue to polymerize there, leading to growing bundles of MTs with minus ends that are pushed away from the kinetochores [21]. These growing bundles, which will give rise to the k-fibers, interact with the astral MTs and eventually coalesce to form a bipolar spindle [21,30]. A similar model applies to both *Drosophila* and human cells that form bipolar spindles in the absence of centrosomes or reassemble a spindle after MT depolymerization. However, in these cases, the kinetochore-driven k-fibers coalesce at the spindle poles through a centrosome-independent mechanism that exploits MT minus end-directed motors and minus end binding proteins [8,30].

Studies on human cells, *Xenopus*-derived systems and *Drosophila* identified several proteins that control chromatin/kinetochore-driven MT growth. For example, this process is inhibited by depletion of TPX2, HURP, Aurora A, Aurora B, or INCENP, whose deficiency does not impair MT nucleation from the centrosomes [22,23,31,32,33]. In *Drosophila* embryos, chromosome-associated MT regrowth is prevented by depletion of the HURP homologue Mars, but not by the TPX2 homologue Mei-38 [19]. In *Drosophila* larval brain cells, kinetochore-driven MT regrowth (KDMTR) is inhibited by loss of Misato (Mst), a protein that interacts with the Tubulin Chaperone Protein-1 (TCP-1) complex and the Tubulin Prefoldin complex, which are also required for KDMTR [34,35]. Other factors required for efficient KDMTR in *Drosophila* somatic cells are Ensconsin (Ens), an MT-binding protein homologous to the human MAP7 [36], γ-tubulin, and the Msps (TOGp) MT polymerase [24]. Finally, in mammalian cells, KDMTR is hampered by loss of the MT minus end binding MCRS1–KANSL1–KANSL3 complex [37,38] and by failure of Nup107-160-dependent γ-tubulin recruitment at kinetochores [39].

Another conserved factor that promotes chromatin-induced MT formation is augmin. Augmin is an 8-protein complex that binds the lateral walls of spindle MTs and recruits γ-TuRCs that nucleate additional MTs, “augmenting” the spindle MT density [40,41,42]. In human cells, *Drosophila* S2 cells, and *Drosophila* embryos, augmin is required for chromosome-driven MT formation and efficient assembly of k-fibers [19,24,40,43,44,45,46]. Interestingly, *Drosophila* mutants in the *wac* and *msd1* genes, each of which encodes an augmin subunit, are viable and do not exhibit defective spindles in larval brains. However, flies homozygous for both *cnn* and *msd1* mutations are lethal and display highly aberrant spindles [47]. These results suggest that in *msd1* mutants, there is a reduced chromosome-driven MT generation, which is sufficient for spindle assembly in the presence of MTs nucleated by the centrosomes, but insufficient in the absence of centrosomal MTs.

All these studies indicate that the mechanisms of KDMTR can be dissected using a genetic approach. Here, we use *Drosophila* S2 cells to analyze KDMTR after colcemid or cold treatment. We examine this process in normal cells and in cells subjected to RNAi-mediated depletion of different evolutionarily conserved proteins. Specifically, we analyze KDMTR in cells depleted of (i) proteins that have been already implicated in KDMTR such as Mars (HURP), Mei-38 (TPX2), and the augmin subunit Dgt6 [19,22,23,24,32,33]; (ii) the plus end-associated factors Eb1 (MAPRE1/EB1) and Mast (CLASP1) (reviewed in [48]) (*mast*/*orbit*, whose official FlyBase name is *chromosome bows*, *chb*, will be henceforth designated as *mast*); (iii) the minus end binding Patronin (CAMSAP2) and Asp (ASPM) (reviewed in [49]); and (iv) the Klp10A kinesin-like protein (KIF2A) that depolymerizes the MT minus ends (reviewed in [50]). We show that depletion of some of these proteins (Mast, Mars, Mei-38, Dgt6, and Eb1) downregulates KDMTR. In contrast, loss of Asp or Klp10A leads to an increased mass of regrowing MTs compared to control. We also examined the localization of GFP-tagged Mast, Mars, Mei-38, Eb1, Patronin, and Asp during KDMTR. This analysis revealed that these proteins exhibit different localizations during KDMTR, which are likely to reflect their specific roles in the process. Collectively, our results define the modes of spindle reassembly after colcemid-induced MT depolymerization and identify proteins that either enhance or reduce KDMTR.

## 2. Materials and Methods

### 2.1. Cell Cultures

The S2 cells used for colcemid-induced MT depolymerization were grown in 39.4 g/L Shields and Sang M3 Insect medium (Sigma-Aldrich, Saint Louis, MO, USA) supplemented with 0.5 g/L KHCO_3_ and 20% heat-inactivated FBS (Gibco Inc., Grand Island, NY, USA). The S2 cells used for cold-induced MT depolymerization were grown in Schneider’s medium (Sigma-Aldrich) supplemented with 10% heat-inactivated FBS (Thermo Fisher Scientific, Waltham, MA, USA). S2 cells expressing GFP-tagged proteins, including cells expressing GFP-tubulin (obtained from the Drosophila Genomics Resource Center, Department of Biology: Indiana University Bloomington, USA) [51], were cultured in 39.4 g/L Shields and Sang M3 Insect medium (Sigma-Aldrich) supplemented with 2.5 g/L bacto peptone (BD, Sparks, MD, USA), 1 g/L yeast extract (Thermo Fisher Scientific), and 5% heat-inactivated FBS (Gibco). All cells were cultured at 25 °C and were free from mycoplasma contamination.

### 2.2. dsRNA Production and RNAi Treatments

The DNA templates for the synthesis of dsRNAs for RNAi against *mast*, *mei-38*, *mars*, *dgt6*, *Eb1*, *Patronin*, *asp*, and *Klp10A* were amplified from wild-type (*Oregon-R*) genomic DNA or cDNA libraries made from 0–24 h *Oregon-R* embryos. The primer sequences used for amplification are reported in Table S1. The PCR products were purified using spin columns. Synthesis of dsRNAs was carried out as described earlier [52].

RNAi treatments were carried out as follows. 1 × 10^6^ S2 cells in 1 mL of the appropriate serum-free medium were plated in a well of a six-well culture plate. A total of 60–80 µg of dsRNA was then added to each well. After 1 h incubation, 2 mL of the FBS-supplemented medium was added, and cells were grown for 5 days. For *Patronin* and *Klp10A* RNAi, a second dose (60–80 µg) of dsRNA was added to the samples on day 3, and cells were grown for 2 additional days. Control S2 cells were prepared in the same way, but without addition of dsRNA.

### 2.3. Evaluation of the RNAi Efficiency by RT-qPCR

Gene-specific primers (different from those used for dsRNA production) were designed using Primer-BLAST (https://www.ncbi.nlm.nih.gov/tools/primer-blast/; accessed on 2 April 2022) or Primer3 (http://bioinfo.ut.ee/primer3-0.4.0/primer3/; accessed on 2 April 2022) software; primer sequences are provided in Table S2. For each primer pair, the amplification efficiency was determined from the slope of the log-linear portion of the calibration curve [53] generated using dilutions of cDNA prepared from wild-type S2 cells (Table S2). Total RNA was isolated from control and dsRNA-treated S2 cells using RNAzol RT reagent (MRC, Cincinnati, OH, USA) according to the manufacturer’s instructions. Reverse transcription was performed with the RevertAid reverse transcriptase (Thermo Fisher Scientific) using 2 µg of total RNA in the presence of 2 U/µL of RNaseOut Recombinant RNase Inhibitor (Thermo Fisher Scientific). Genomic DNA was eliminated using the RapidOut DNA Removal Kit (Thermo Fisher Scientific). qPCR was carried out using BioMaster HS-qPCR SYBR Blue (2×) reagent kit (Biolabmix; Novosibirsk, Russia; https://biolabmix.ru/en/; accessed on 2 April 2022) and CFX96 Real-Time PCR Detection System (Bio-Rad, Hercules, CA, USA). We used the following thermal cycling conditions: 5 min at 95 °C, followed by 39 cycles of 15 s at 95 °C, 30 s at 60 °C, and 30 s at 72 °C. Data were collected during each extension phase. Negative control templates (water and cDNA synthesized without reverse transcriptase) were included in each run. Measurements of gene expression were performed in at least four biological replicates, each with three technical replicates. The relative mRNA quantification was determined using the ΔΔCq method [53]. mRNA expression levels were normalized to those of the housekeeping gene *RpL32*; primers for this gene are from [54]).

### 2.4. Colcemid-Induced MT Depolymerization

Control and RNAi samples were incubated for 3 h in a medium containing colcemid (Calbiochem, Merck, KGaA, Darmstadt, Germany) at the final concentration of 4.5 µg/mL. After incubation, cells were centrifuged (1000 *g*, 22 °C, 5 min) and the colcemid-containing medium was removed. Cells were washed three times in drug-free medium and left in this medium. Cells were then fixed after 20–75 min after colcemid removal or handed over for in vivo observation by confocal microscopy (cells expressing GFP-tagged proteins). A part of the cells was fixed at the end of 3 h colcemid treatment (without removal of the drug) to check the degree of MT depolymerization (time 0).

### 2.5. Cold-Induced MT Depolymerization

For cold-induced MT depolymerization, both control and RNAi samples were incubated on ice for 3 h. Cells were then returned to 22 °C and fixed at different times after exposure to this temperature. Some cells were fixed at the end of the 3 h cold treatment (before exposure to 22 °C) to verify the degree of MT depolymerization (time 0).

### 2.6. Generation of S2 Cells Expressing GFP-Tagged Proteins

S2 cell lines expressing mCherry-αTub84B (referred to as Cherry-tubulin throughout the manuscript) and either Asp-GFP or Patronin-GFP, were generated previously [55]. The other cell lines expressing both Cherry-tubulin and GFP-tagged proteins used here were generated using a piggyBac transposon-based vector as described in [52,55]. Full-length coding sequences of *Eb1*, *mars*, *mast*, and *mei-38* were cloned in the piggyBac transposon vector in frame with the enhanced GFP (referred to as GFP throughout the manuscript) coding sequence and under the control of the copper-inducible *MtnA* promoter. The resulting plasmid constructs, verified by sequencing, encode the C-terminal GFP fusions of Eb1 (GenPept accession no. AGB93267.1), Mars (GenPept accession no. AHN56186.1), Mast (GenPept accession no. AAN12151.1), or Mei-38 (GenPept accession no. AAF45636.1, but with V99D, A117D, S120P, and E162_R163insA mutations, caused by known DNA sequence variations (http://dgrp.gnets.ncsu.edu/; accessed on 2 April 2022); [56,57]). Each plasmid also contained the Cherry-tubulin coding sequence under the control of the *MtnA* promoter and a blasticidin-resistance cassette. Details of the plasmid constructions are available upon request. Integration of the transgenes in the genome of S2 cells and the subsequent selection of modified cells with blasticidin were performed as described in [52]. All cells were free from mycoplasma contamination. To induce expression of fluorescent fusion proteins, cells were grown in the presence of 100–250 µM of copper sulfate for 16–22 h before in vivo analysis or fixation.

### 2.7. Fixation, Immunostaining, and Microscope Analysis

For mitotic phenotype analysis, cells were fixed as described in [58]. Briefly, 2 × 10^6^ S2 cells were spun down by centrifugation at 1000 *g* for 5 min, washed in 2 mL of 1 × PBS (Sigma), and fixed for 10 min in 2 mL of 3.7% formaldehyde in 1 × PBS. Cells were resuspended in 500 µL of PBS, and cytocentrifuged on clean slides (using a Cytospin 4 Cytocentrifuge, Thermo Fisher Scientific, at 900 rpm for 4 min). Slides were then immersed in liquid nitrogen for 5 min, transferred to PBT (PBS plus 0.1% TritonX-100) for 30 min, and then to PBS containing 3% BSA (AppliChem, GmbH, Darmstadt, Germany) for 30 min.

The slides were immunostained using the following primary antibodies, all diluted in a 1:1 mixture of PBT and 3% BSA: mouse anti-α-tubulin (1:500, Sigma-Aldrich, T6199), rabbit anti-Spd-2 (1:4000, [15]), rabbit anti-Cid (1:300, Abcam Inc., Eugene, Oregon, USA, ab10887), and chicken anti-GFP (1:200, Thermo Fisher Scientific, PA1-9533). Primary antibodies were detected by incubation for 1 h with FITC-conjugated goat anti-mouse IgG (1:40, Sigma-Aldrich, F8264) or Alexa Fluor 568-conjugated goat anti-mouse IgG (1:300, Thermo Fisher Scientific, A11031), Alexa Fluor 568-conjugated goat anti-rabbit IgG (1:350, Thermo Fisher Scientific, A11036) or Alexa Fluor 660-conjugated goat anti-rabbit IgG (1:300, Thermo Fisher Scientific, A21074), and Alexa Fluor 488-conjugated goat anti-chicken IgG (1:300, Thermo Fisher Scientific, A11039). To reduce fluorescence fading, most slides were mounted in Vectashield antifade mounting medium containing 4.6-diamidino-2-phenylindole (DAPI; Vector Laboratories, Newark, CA, USA). Slides stained with Alexa Fluor 660 were stained with DAPI and then mounted in the ProLong Gold Antifade Mountant (Thermo Fisher Scientific). Images of fixed cells were captured using a Zeiss Axio Imager M2 equipped with an EC Plan-Neofluar 100×/1.30 oil lens (Carl Zeiss Microscopy, Jena, Germany) and with an AxioCam 506 mono (D) camera. Some of the fixed cell images were generated using a Zeiss LSM 710 confocal microscope and an oil immersion 63×/1.40 plan-apo lens with an AxioCam 506 mono camera using the ZEN 2012 software (Carl Zeiss Microscopy). All slides were coded and blindly scored. The patterns of KDMTR at 30 min after colcemid removal and at 30 s after cold treatment (see Figures below) were determined by examining microphotographs.

For live cell analysis, 500 μL of cell suspensions (5 × 10^5^ cells/mL) were transferred to cell chambers (Invitrogen A-7816) containing coverslips treated with 0.25 mg/mL concanavalin A (Sigma-Aldrich C0412) placed on the bottom of the chambers. In some cases, live cells were treated with Hoechst 33342 (Thermo Fisher Scientific, H3570, final concentration 20 μg/mL) to stain chromosomal DNA. Observations were performed between 20 and 180 min after cell plating using the same Zeiss confocal microscope described above.

### 2.8. Spindle Length Measurements

The spindle length was measured with the ZEN 2012 software (Carl Zeiss Microscopy) as described in detail in [52]. Measures were restricted to cells that did not appear to be polyploid with respect to the basic karyotype of S2 cells. The data were compared using the Mann–Whitney *U* test and plotted using the BoxPlotR web-tool (http://shiny.chemgrid.org/boxplotr/; accessed on 2 April 2022) [59].

### 2.9. Fluorescence Intensity Measures

The chromosome-associated tubulin fluorescence (CATF) intensity of regrowing MTs after colcemid-induced MT depolymerization was measured with the Fiji program [60] using the “Freehand selections” tool and the function “Measure”. Microphotographs in Carl Zeiss Image (CZI) format captured by a Zeiss Axio Imager M2 were used. In all cases, we encircled the tubulin signals that were clearly associated with the kinetochores/chromosomes and measured them individually. To calculate the CATF of prometaphases and metaphases (PRO-METs), we subtracted the background fluorescence from each tubulin signal and then summed all values. To make comparable different experiments, we normalized the fluorescence intensity of each RNAi and control PRO-MET from the same biological replicate to the mean fluorescence intensity value of the control cells.

## 3. Results

To investigate the mechanisms of KDMTR, we used a reverse genetic approach. We performed RNAi in *Drosophila* S2 cells against 8 genes encoding proteins required for mitotic spindle assembly and then examined RNAi cells for spindle reformation after MT depolymerization with colcemid. Specifically, we focused on *mast*, *mars*, *mei-38*, *Eb1*, *dgt6*, *Patronin*, *asp*, and *Klp10A.* Before performing the KDMTR experiments, we checked the efficiency of RNAi by quantifying the reduction of target mRNA level and examining the phenotypic consequences thereof.

### 3.1. Efficiency of RNAi

**Figure 1:**
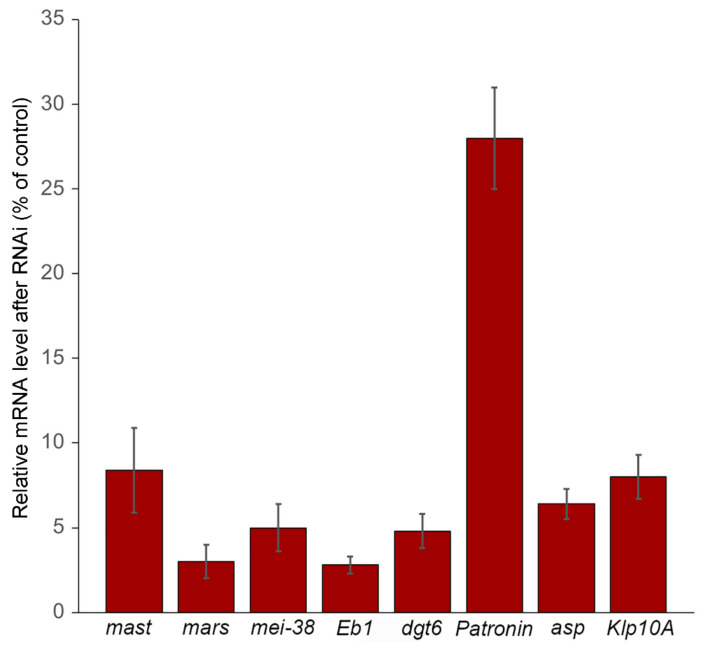
Relative *mast*, *mars*, *mei-38*, *Eb1*, *dgt6*, *Patronin, asp*, and *Klp10A* mRNA levels observed after RNAi against these genes. The analysis was performed by RT-qPCR and reported as the median values ± SEM relative to control. Data are from at least four independent experiments.

We determined the level of target mRNA after 5 days incubation with the corresponding double-stranded RNA (dsRNA). In all cases (except *Patronin*), we found mRNA levels ranging from 3 to 8% of the untreated cells level; *Patronin* mRNA was reduced to 28% of control (Figure 1). To check the effectiveness of mRNA depletion, we also analyzed the mitotic phenotypes of RNAi cells. We examined fixed RNAi cells immunostained for α-tubulin and the centrosome marker Spd-2 and counterstained for DNA with DAPI. Consistent with previous results [55,61,62,63], we found that RNAi-mediated depletion of the Klp10A MT depolymerase or the Asp minus end binding protein results in longer late prometaphase/metaphase spindles compared to those observed in control cells (Figures S1 and S2A). Klp10A accumulates at the kinetochores, the centrosomes, and the spindle poles but is thought to act mainly at the poles [64,65]. *Klp10A* RNAi cells showed frequent monopolar spindles and a modest but significant increase in the frequency of prometaphase-like cells with elongated spindles (PMLES) (Figures S1 and S2B; see also [66]). PMLES (or pseudo ana-telophases) have been previously observed in cells where the kinetochore–MT interaction was compromised, such as those depleted of the centromeric histone Cid (CENPA) or the Ndc80 kinetochore protein [17,67]. Asp binds the MT minus ends [68] and is enriched at both the spindle poles and the extremities of the central spindle [55,62,69,70]. In agreement with these studies, we found that Asp-depleted cells exhibit broad spindle poles that often show centrosome detachment, and defects in chromosome alignment and segregation (i.e., PMLES; Figures S1 and S2B).

Cells depleted of Mast, Mars, Mei-38, Dgt6, Eb1, or Patronin are all characterized by spindles significantly shorter than those of control cells (Figures S1 and S2A; see also [24]) but exhibit very different mitotic phenotypes. The most dramatic phenotype was observed in cells depleted of Mast, which mediates the incorporation of tubulin dimers into the plus ends of the MTs embedded in the kinetochores [71]. As described previously [72,73], in *mast* RNAi cells, most spindles collapse forming monopolar figures, while the short bipolar spindles show defective chromosome alignment and segregation (Figures S1 and S2B). Eb1 is enriched at the plus ends of growing MTs and its depletion results in short and malformed spindle and defective chromosome segregation [51]. In line with these results, we found that *Eb1* RNAi cells exhibit many morphologically abnormal spindles and a higher frequency of PMLES compared to control (Figures S1 and S2B). Frequent PMLES and monopolar spindles were also observed in cells depleted of the augmin component Dgt6, as described previously [24]. Depletion of Patronin, which binds the MT minus ends, resulted in short spindles often associated with multiple centrosomes (Figures S1 and S2B; see also [55]). The spindles of both *mei-38* and *mars* RNAi cells were shorter than control spindles but did not show gross morphological defects, in agreement with previous results [74,75,76]. However, *mei-38* RNAi cells displayed an increase in monopolar spindles compared to control. A small but significant increase in monopolar spindles was also observed in *mars* RNAi cells, together with mild defects in chromosome segregation (Figures S1 and S2B).

### 3.2. KDMTR after Colcemid-Induced MT Depolymerization

To address the roles of the genes of interest in KDMTR, we analyzed the effects of their RNAi-mediated downregulation following colcemid-induced MT depolymerization. Previous work has shown that after cold-induced MT depolymerization, the centrosomes of S2 cell prometaphases and metaphases (PRO-METs) are normally placed at the presumed location of the depolymerized spindle poles, where they nucleate MT asters [24,77]. In contrast, after colcemid treatment, the centrosomes are no longer able to drive astral MT regrowth and in most cells are freely floating in the cytoplasm ([77]; see also below). As a result, in many cases, one or both poles of the reformed spindles were not associated with the centrosomes [77]. Thus, by analyzing MT regrowth after colcemid-treatment, we are in fact studying KDMTR in the absence of centrosomal activity.

Control and RNAi (*mast*, *mars*, *mei-38*, *Eb1*, *Patronin*, *dgt6*, *asp*, and *Klp10A*) cells were treated for 3 h with colcemid. Cells were then accurately washed to remove colcemid and fixed after 20, 30, 45, and 75 min after removal of the drug. Some cells were fixed after 3 h of colcemid treatment without removal of the drug, to check the degree of MT depolymerization (time 0). All cells were immunostained for both α-tubulin and the centrosomal marker Spd-2 and counterstained with DAPI. We limited our observations to PRO-METs, as the kinetochores of cells in these mitotic phases have the ability to drive k-fiber formation and bipolar spindle reassembly [77].

**Figure 2:**
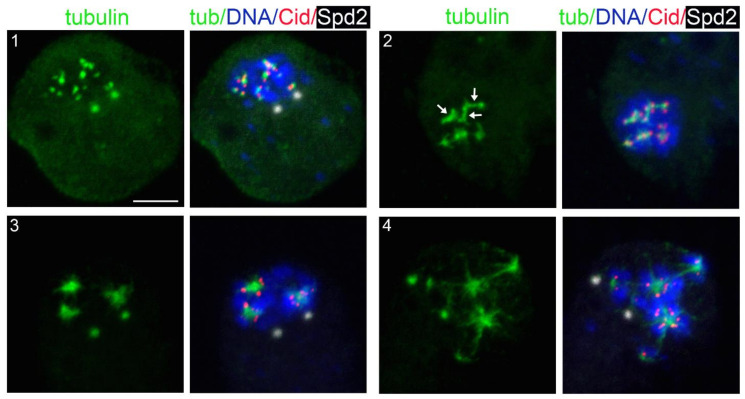
Representative examples of kinetochore-driven microtubule regrowth (KDMTR) figures after colcemid-induced MT depolymerization. S2 cells were fixed and stained with anti-Cid (red), anti-α-tubulin (green), and anti-Spd-2 (white) antibodies and counterstained for DNA (DAPI, blue). (**1**) Shows an initial MT regrowth phase with short MT bundles associated with centromeric Cid signals that mark kinetochore positions. Note that these bundles, collectively designated as “short bundles”, vary in size depending on whether they emanate from one or two sister kinetochores. In (**2**), some of the initial MT bundles have elongated and appear as relatively long MT bundles (arrows), hereafter designated as “long MT bundles”. (**3**,**4**) Illustrate a further regrowth phase, with MTs forming aster-like structures (henceforth “MT clusters/asters”), which are surrounded by centromeres. Scale bar, 5 µm.

**Figure 3:**
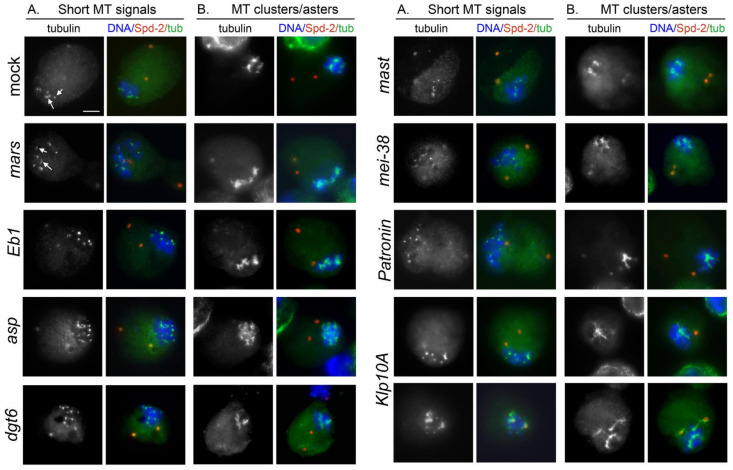
Examples of representative kinetochore-driven microtubule regrowth (KDMTR) figures in mock-treated and RNAi cells following colcemid-induced (3 h, 4.5 μg/mL) MT depolymerization. S2 cells were fixed 30 min after colcemid washout and stained for α-tubulin (green) and centrosomes (Spd-2, red), and counterstained for DNA (DAPI, blue). The cells show several combinations of KDMTR figures: (i) short MT bundles; (ii) long MT bundles; (iii) MT cluster/asters. The A panels show the initial phases of MT regrowth, characterized by the presence of many short MT bundles (short arrows) and a few long bundles (long arrows). The B panels show more advanced stages of regrowth marked by the presence of MT clusters/asters. Note that after colcemid treatment, the prometaphase and metaphase chromosomes of both control and RNAi cells remain clustered, while in most cases, the centrosomes are not associated with regrowing MTs and appear to float freely in the cytoplasm. In contrast, in a substantial fraction of *Klp10A* RNAi cells the centrosomes are surrounded by MTs. Control and RNAi cells show morphologically comparable KDMTR figures but differ in the frequency of these figures, as well as in the fluorescence intensity of regrowing MTs (see Figure 5 and Figure 6 below). Scale bar, 5 µm.

**Figure 4:**
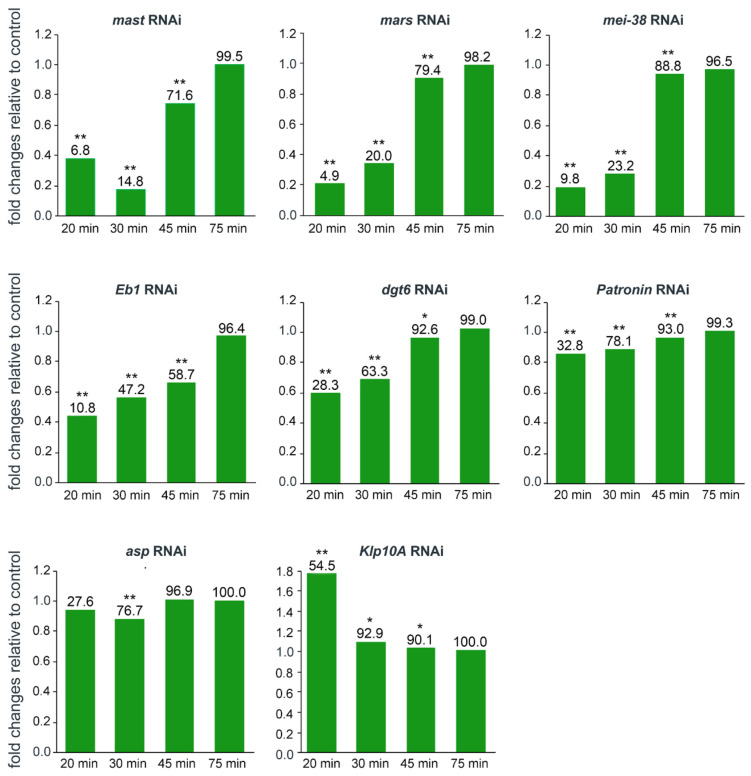
Analysis of the changes in the frequencies of cells showing kinetochore-driven microtubule regrowth (KDMTR) after RNAi against the indicated genes. The columns show the fold changes relative to control at different times after colcemid washout. We considered KDMTR-positive any PRO-MET showing at least one chromosome-associated bright tubulin signal (see examples in Figure 2 and Figure 3). The control is set to 1. * and ** indicate significance in the χ^2^ test with *p* ≤ 0.05 and ≤ 0.01, respectively. The numbers on top of each column indicate the actual frequency (%) of PRO-METs showing KDMTR. In all cases, except *Klp10A* RNAi, data are from at least three independent experiments; for *Klp10A* RNAi cells, data are from two independent experiments. In each experiment, we made slides from mock-treated control cells and RNAi cells (we usually performed RNAi against two different genes in parallel). The fold changes are relative to the specific internal control of each experiment and not to the average value of all control experiments. The total numbers of RNAi and control PRO-METs scored for each experiment at different fixation times were as follows (# RNAi cells/# control cells). *mast*: 20 min 713/724, 30 min 725/731, 45 min 512/525, 75 min 202/207; *mars*: 20 min 1045/1030, 30 min 973/972, 45 min 842/861, 75 min 613/619; *mei-38*: 20 min 707/714, 30 min 714/715, 45 min 510/516, 75 min 200/209; *Eb1*: 20 min 866/842, 30 min 617/623, 45 min 413/427, 75 min 415/448; *dgt6*: 20 min 810/816, 30 min 721/832, 45 min 404/404, 75 min 404/420; *Patronin*: 20 min 830/865, 30 min 770/830, 45 min 579/619, 75 min 594/638; *asp*: 20 min 847/835, 30 min 630/626, 45 min 434/420, 75 min 419/419; *Klp10A*: 20 min 200/205, 30 min 208/401, 45 min 410/411, 75 min 408/408.

At time 0, in more than 95% of both control and RNAi PRO-METs the spindle was completely depolymerized, while ~5% showed only some weakly fluorescent remnants of the spindle MTs. These results indicate that none of the RNAi treatments affect colcemid-induced MT depolymerization. Cells collected at different times after colcemid removal showed different frequencies of PRO-METs with KDMTR. We considered as KDMTR-positive the PRO-METs that exhibit at least one very bright kinetochore-associated tubulin signal; examples of initial tubulin regrowth signals are shown in Figure 2 and Figure 3. At 20, 30, and 45 min after colcemid removal in *mast*, *mars*, *mei-38*, *Eb1*, and *Patronin* RNAi cells, the frequencies of PRO-METs showing KDMTR were significantly lower than in control (Figure 4). *dgt6* RNAi cells with KDMTR were less frequent than in control only at the 20 and 30 min fixation times (Figure 4). In *asp* and *Klp10A* RNAi cells, the frequencies of PRO-METs showing KDMTR were broadly similar to the control frequencies with two exceptions. At 20 min, the frequency of *Klp10A* RNAi cells showing KDMTR was higher than in control, while at 30 min, Asp-depleted cells with KDMTR were slightly but significantly less frequent than in control (Figure 4). After 75 min of MT regrowth, nearly all RNAi and control cells displayed KDMTR (Figure 4). These results suggest that cells depleted of Mast, Mars, Mei-38, Eb1, Patronin, or Dgt6 are defective in KDMTR. However, we would like to note that the frequency of cells showing KDMTR reflects the capability of mitotic cells to initiate but not to sustain and promote this process during spindle reassembly.

To gather additional information about KDMTR, we analyzed the pattern of MT regrowth. After colcemid-induced MT depolymerization, MT repolymerization in control cells started at kinetochores, which became tightly associated with small dot-like tubulin signals (Figure 2). These tubulin dots then expanded, forming tubulin bundles and tubulin aggregates that often appeared as aster-like structures. However, these structures did not contain centromeres at their centers but were instead surrounded by centromere signals (Figure 2). In some cells, centrosomes were associated with weak tubulin signals (Figure 2 and Figure 3) but never showed astral MT regrowth, as occurs after cold-induced MT depolymerization ([24,77]; see also below). We distinguished three types of KDMTR figures: (1) kinetochore-associated tubulin dots and double dots (or rods of the size of a double dot) corresponding to the initial MT regrowth from a single kinetochore or both sister kinetochores, collectively designated as “short MT bundles” (Figure 2 and Figure 3); (2) MT bundles resulting from the elongation of the “double dots”, designated as “long MT bundles” (Figure 2 and Figure 3); (3) “MT clusters/asters” resulting from the association and overlapping of regrowing MT bundles (Figure 2 and Figure 3). The MT clusters/asters then coalesced and emanated very long MT bundles extending towards the spindle poles (hereafter, “extended MT bundles”), resulting in different types of MT intermediate arrangements, including many umbrella-like formations in which the regrowing bundles converge on one side and diverge on the other. Most likely, each of these intermediate figures will give rise to a bipolar anastral spindle. For example, as documented in previous studies (see for example [16,78]), the divergent MT bundles of the umbrella-like structures elongate and progressively merge to form a second spindle pole. Reformation of a bipolar spindle after colcemid-induced MT depolymerization is shown in Figure S3; the structure of the MT clusters/asters and the formation of extended MT bundles are described below together with Asp-GFP and Mast-GFP localization during spindle reassembly.

**Figure 5:**
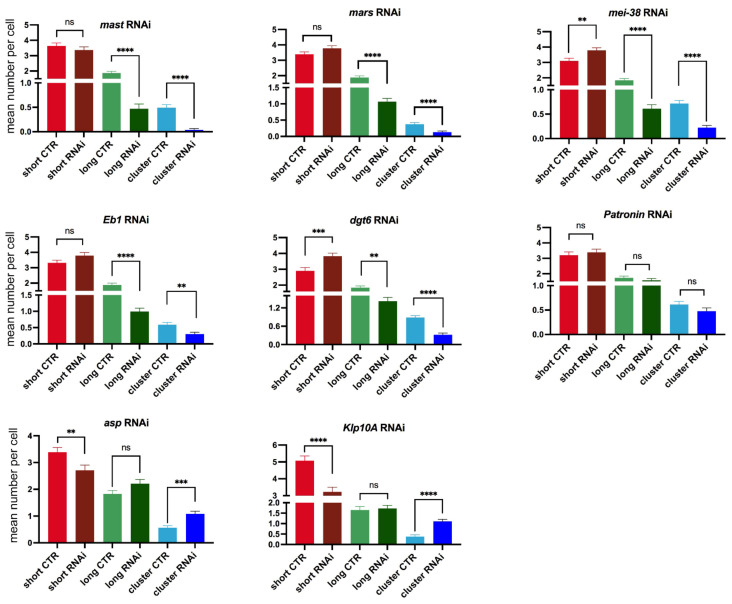
Mean numbers per cell of the indicated kinetochore-driven microtubule regrowth (KDMTR) figures (short MT bundles, long MT bundles and MT clusters/asters) observed 30 min after colcemid washout. The mean numbers (±SEM) of the different KDMTR figures were determined by examining only cells that exhibit KDMTR. The maximum number of clusters/asters assigned to each cell was three. In all cases, except *Klp10A* RNAi, data are from at least three independent experiments; for *Klp10A* RNAi cells data are from two independent experiments. The numbers of RNAi and control cells examined for definition of the KDMTR pattern are as follows (# of RNAi cells/# of control cells): *mast*, 81/120; *mars*, 150/183; *mei-38*, 126/147; *Eb1*, 119/148; *dgt6*, 138/144; *Patronin*, 113/124; *asp*, 123/143; *Klp10A*, 94/86. The data were analyzed with the Mann–Whitney *U* test; ns, not significant; **, ***, **** significant with *p* ≤ 0.01, 0.001, and 0.0001, respectively.

At different fixation times, control cells showed different frequencies of short and long MT bundles and MT clusters/asters (Figure S4). At 20 min, the cells with KDMTR were relatively few and showed very small tubulin signals (mostly short MT bundles). At 30 and 45 min fixation times, the PRO-METs displayed short and long MT bundles and MT clusters/asters. However, at 45 min, the MT clusters/asters were more frequent and larger than at 30 min and often had complex morphologies. At 75 min, 60% of the PRO-METs showed partially or completely reformed spindles, while in the remaining 40%, the chromosomes were associated with large clusters/asters (Figure S4). Thus, to quantitate KDMTR in control and RNAi cells we focused on PRO-METs fixed 30 min after colcemid removal, as these cells exhibit clear kinetochore-associated tubulin signals that can be examined for morphology. Importantly, we found that control and RNAi PRO-METs exhibit very similar KDMTR figures; however, they vary in frequency according to the RNAi treatment. As mentioned above, we distinguished three types of MT regrowth figures: short and long kinetochore-associated MT bundles and MT clusters/asters. As shown in Figure 2 and Figure 3, MT clusters/asters are usually well-separated. Large clusters/asters presumably resulting from the overlap of two asters were considered as a single aster, and no more than three clusters/asters were assigned to cells showing multiple asters. In *mast*, *mars*, *mei-38*, *dgt6*, and *Eb1* RNAi cells, the frequencies per cell of long MT bundles and MT clusters/asters, were significantly reduced compared to controls (Figure 5). In Patronin-depleted cells, the frequencies of long MT bundles and clusters/asters were slightly but not significantly reduced (Figure 5). In contrast, Asp- and Klp10A-depleted cells showed significant increases in MT clusters/asters compared to controls (Figure 5). The effect of Asp depletion on KDMTR was completely unexpected and suggests a role for this protein in the process of kinetochore-driven MT growth in unperturbed cells.

Interestingly, in Klp10A-deficient cells, a large fraction (55%) of the centrosomes were associated with strong tubulin signals (Figure 3). Notably, the centrosomes of *Klp10A* RNAi cells showed the same levels of Spd-2 as both control cells and other RNAi cells. This suggests that the ability of *Klp10A* RNAi cells to drive MT regrowth after colcemid treatment is not dependent upon variation in the levels of pericentriolar material (PCM) proteins such as Spd-2. One possibility is that the centrosomes of colcemid-treated cells retain some MT nucleating ability and that the regrowing MTs are particularly sensitive to the Klp10A MT depolymerase. Alternatively, colcemid-treated centrosomes might have lost the ability to shield the minus ends of the MTs they nucleate from the activity of Klp10A. Regardless of the explanation, our findings underlie an interesting aspect of centrosome biology, which deserves future studies.

**Figure 6:**
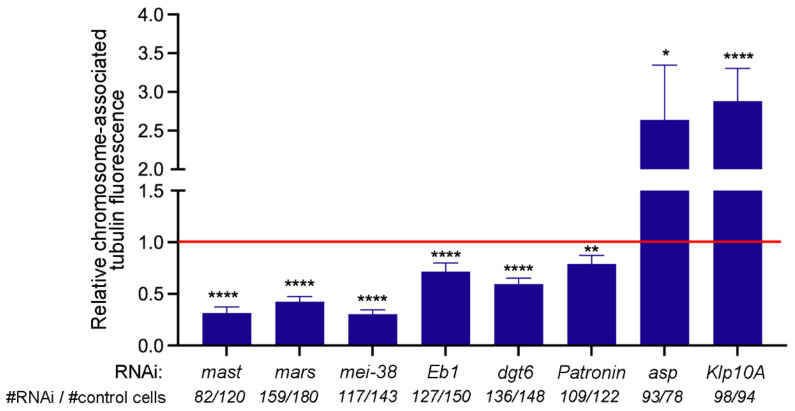
Mean fold changes relative to control of the tubulin fluorescence intensity of cells showing kinetochore-driven microtubule regrowth (KDMTR). The mean fold changes (±SEM) of chromosome-associated tubulin fluorescence (CATF) relative to control (set to 1; red line) were determined by examining only PRO-METs that exhibit KDMTR. In all cases, except *Klp10A* RNAi, data are from at least three independent experiments; for *Klp10A* RNAi cells, data are from two independent experiments. The total numbers of RNAi and control PRO-METs (# RNAi cells/# control cells) examined for each experiment are indicated on the bottom of the graph. The data were analyzed with the Mann–Whitney *U* test; ns, not significant; *, **, **** significant with *p* ≤ 0.05, 0.01, and 0.0001, respectively.

To provide further support to our observations on the MT regrowth patterns in RNAi cells, we measured the chromosome-associated tubulin fluorescence (CATF). This analysis was performed only on cells that showed KDMTR. Namely, we examined cells displaying at least one bright kinetochore-associated tubulin signal, while cells with no clear KDMTR signals were not included in the analysis (see Figure 4 for the frequencies of RNAi cells showing KDMTR). Consistent with our analyses of morphological classes of MT regrowth (Figure 5), in *mast*, *mars*, *mei-38*, *dgt6*, and *Eb1* RNAi cells, the mean CATF was significantly reduced compared to controls (Figure 6). In Patronin-depleted cells, which showed a MT regrowth pattern comparable to control (Figure 5), the mean CATF was also significantly lower than in control cells (Figure 6). Finally, Asp- and Klp10A-depleted cells showed significant increases in both MT clusters/asters (Figure 5) and CATF (Figure 6) compared to controls. These results indicate in all cases, except *Patronin* RNAi, that the variations in the frequencies of MT clusters/asters correlate well with the mean CATF. This correlation, however, is not found in *Patronin* RNAi cells, where a significant reduction in CATF does not correspond to a concomitant reduction in the frequency in MT cluster/aster. This suggests that the regrowing MT structures observed in Patronin-depleted cells might contain fewer MTs that those of control cells.

### 3.3. KDMTR after Cold-Induced MT Depolymerization

**Figure 7:**
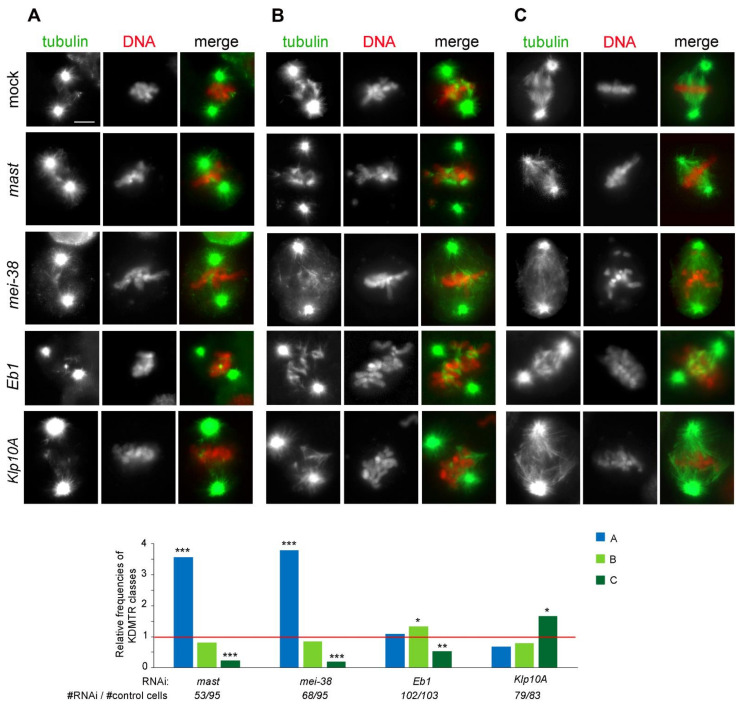
Types and frequencies of MT regrowth figures in mock-treated and RNAi cells following cold-induced (3 h at 0 °C) MT depolymerization. The top panels show representative examples of PRO-METs fixed 30 s after their return to 22 °C and stained for α-tubulin (green) and DNA (red). According to the patterns of chromosome-associated MT regrowth, PRO-METs are subdivided into three classes, showing (**A**) from one to four short MT bundles; (**B**) randomly oriented long MT bundles; (**C**) long MT bundles with a chromosome-to-pole orientation as in a normal spindle. Control and RNAi cells show qualitatively similar KDMTR figures but differ in the frequency of these figures. Scale bar, 5 µm. The graph shows the frequencies of the KDMTR classes relative to control (set to 1; red line). Data are from at least two independent experiments; the numbers of RNAi and control cells examined (# of RNAi cells/# of control cells) are indicated on the bottom of the graph. *, **, ***, significant in the χ^2^ test with *p* ≤ 0.05, 0.01, and 0.001, respectively.

We next wondered whether the observed pattern of MT regrowth and spindle reassembly was a specific outcome of colcemid-induced MT depolymerization or was instead independent from the procedure used to depolymerize MTs. To address this question, we examined cells recovering from cold-induced MT depolymerization. We limited our observations to mock-treated cells and cells subjected to RNAi against four representative genes (*mast*/*orbit*, *mei-38*, *Eb1*, and *Klp10A*). RNAi and untreated control cells were incubated at 0 °C for 3 h, placed at 22 °C, and then fixed at various times after returning to 22 °C. Cell samples were also fixed before they were transferred to 22 °C (time 0). All cells were then immunostained for both α-tubulin and Spd-2 and counterstained with DAPI. In both control and RNAi cells, MT regrew from both the centrosomes and the kinetochores, which were normally placed at the presumed location of the depolymerized spindle poles (Figure 7) and not floating in the cytoplasm as after colcemid treatment (see Figure 3; [77]).

At time 0 in more than 90% of control and RNAi cells, the spindle was completely depolymerized; only in a few cells we observed dull tubulin fluorescence associated with the centrosomes or the remnants of the k-fibers, indicating that none of the RNAi treatments substantially affect the process of cold-induced depolymerization of the spindle MTs. At 20 s fixation time, some of the kinetochore-associated tubulin signals were too weak to be reliably assessed, while in cells fixed at 40 and 60 s the centrosome- and the kinetochore-associated tubulin signals were often overlapping. Thus, we focused on the cells fixed 30 s after return to 22 °C, as these cells exhibit clear kinetochore-associated tubulin signals that can be classified according to their morphology (Figure 7).

Analysis of PRO-METs fixed at 30 s showed that control and RNAi cells display very similar chromosome-associated MT regrowth figures; as for colcemid-treated cells, we did not identify any RNAi-specific KDMTR pattern. In both control and RNAi cells, we observed three classes of PRO-METs showing chromosome-associated bright tubulin signals: (i) PRO-METs with randomly oriented and well-separated short MT bundles (class A; we included in class A cells showing no more than four short, nonoverlapping MT bundles; Figure 7), (ii) PRO-METs with randomly oriented long MT bundles intermingled and overlapped with the chromosomes (class B, Figure 7), and (iii) PRO-METs with long MTs showing a chromosome-to-pole orientation as in a normal spindle (class C; Figure 7). Control and RNAi cells differed in the frequencies of these MT regrowth patterns. Specifically, compared to control, the frequencies of class C cells were strongly reduced in *mast* and *mei-38* RNAi cells and to a lesser extent in Eb1-depleted cells (Figure 7). In contrast, in Klp10A-depleted cells, the frequency of class C cells was significantly increased compared to control (Figure 7). These results indicate that depletion of Mast, Mei-38, or Eb1 negatively regulates KDMTR after colcemid- and cold-induced MT depolymerization, while Klp10A depletion stimulates KDMTR after both depolymerizing treatments. Thus, at least for the four proteins tested here (Mast, Mei-38, Eb1, and Klp10A), the KDMTR process appears to be independent of the method used for MT depolymerization.

### 3.4. Localization of Selected Spindle-Associated Proteins during KDMTR

To investigate the roles of the proteins that regulate KDMTR, we analyzed their localization during the process of spindle regrowth after colcemid treatment. In a previous study on MT regrowth after cold exposure, we showed that the bundles of reforming MTs are uniformly enriched in Dgt6 since the beginning of their formation [24]. Here, we analyzed the localization of Mast, Mars, Mei-38, Eb1, Patronin, and Asp. We generated stable S2 cell lines expressing Cherry-tubulin and each of the proteins of interest marked with GFP, both under the control of a copper-inducible promoter. The lines expressing the GFP fusions of the Patronin and Asp were described in a previous study [55]. The other lines were generated during the present investigation and their precise features are reported in Materials and Methods.

After induction of the transgenes by copper sulfate, Cherry-tubulin and the GFP-tagged proteins were visualized either in living cells or in fixed cells stained with anti-GFP and anti-α-tubulin antibodies and counterstained with DAPI. We found a perfect correspondence between the staining pattern of live and fixed cells, although in some cells tubulin staining was brighter after fixation and immunostaining compared to living cells. In all cases, the localization of GFP-tagged proteins in untreated cells was fully consistent with that observed in previous reports. Mast-GFP was bound to all spindle MTs and specifically enriched at the kinetochores, the spindle poles, and the telophase spindle midzone (Figure S5; see also [79]). Mei-38-GFP was uniformly distributed on the spindle MTs during all mitotic phases (Figure S6; see also [76]). Mars-GFP too was uniformly distributed on PRO-MET spindles, but it was absent from the centrosome area and the telophase central spindle (Figure S7; see also [75,80]). Eb1-GFP was associated with all spindle MTs and enriched at the growing MT plus ends (Figure S8; see also [51]). Asp-GFP localized to all spindle MTs and concentrated at the spindle poles and the extremities of the telophase central spindle that are enriched in MT minus ends (Figure S9; see also [62,69,81]). For Patronin-GFP, we confirmed the peculiar behavior we described previously [55]. In several live prometaphases, Patronin-GFP was associated with the entire spindle that appeared as a weakly and uniformly stained structure. However, these prometaphases suddenly showed brightly fluorescent Patronin-GFP signals associated with short MT bundles located near the chromosomes. These bright signals extended towards the cell poles along preexisting MT bundles (probably k-fibers) and stopped growing just before reaching the poles. Consistent with this finding, fixed prometaphases displayed both GFP-stained and unstained MT bundles [55]. This behavior cannot be a consequence of Patronin-GFP overexpression as both dully fluorescent PRO-METs and those containing the bright MT bundles are likely to contain the same amounts of Patronin-GFP. We hypothesize that when GFP-tagged Patronin binds the k-fibers and moves towards the spindle poles, possibly driven by minus end-directed motors and/or MT flux, it changes conformation exposing its GFP moiety. This conformational change would light up the k-fibers and would also result in a strong reaction with the anti-GFP antibody.

To gather information on the specific roles of the proteins that regulate KDMTR, we examined their behavior during MT regrowth after colcemid-induced depolymerization. The cell lines expressing Cherry-tubulin and the GFP-tagged protein of interest, both under the control of a copper-inducible promoter, were treated for 16–22 h with copper sulfate; exposed to colcemid for 3 h; washed; and then fixed at 30, 45, 90, and 120 min after colcemid removal. Some cells were fixed at the end of colcemid treatment before removal of the drug (time 0). The multiple fixation times permitted us to analyze different stages of MT regrowth, ranging from the early stages mostly consisting of short MT bundles to the late stages containing extended MT bundles often converging into a completely reformed bipolar spindle.

**Figure 8:**
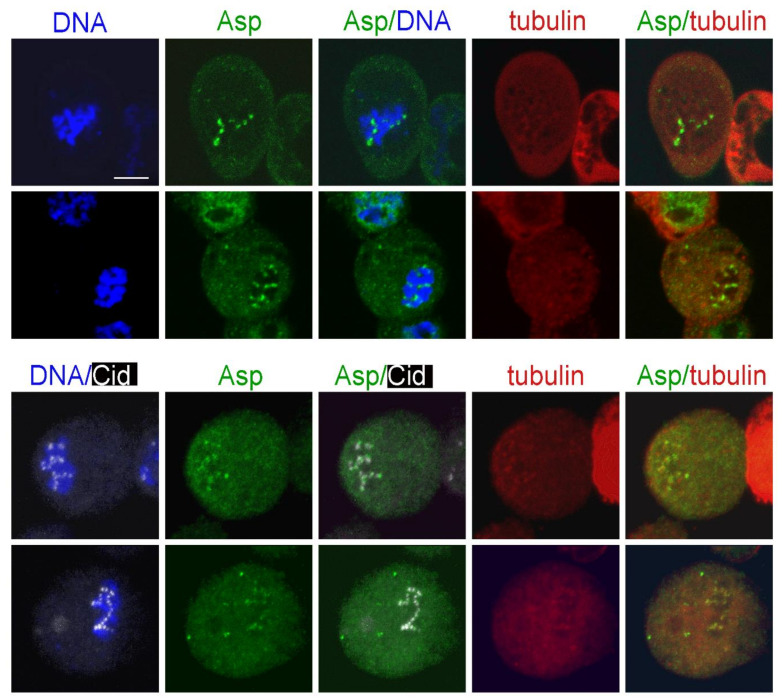
Asp-GFP localization in prometaphases exposed to colcemid for 3 h and photographed before colcemid washout. The top panels show live S2 cells expressing Asp-GFP (green) and Cherry-tubulin (red) stained with the vital DNA stain Hoechst 33342 (blue). The bottom panels show representative fixed S2 cells expressing Asp-GFP stained with anti-GFP antibodies (green), anti-Cid antibodies (white), anti-α-tubulin antibodies (red), and counterstained for DNA (DAPI; blue). Note that Asp-GFP is associated with the kinetochores. Some of the Asp-GFP signals are also associated with formations enriched in Cherry-tubulin. In the Asp/tubulin merges, the fluorescence intensity and contrast of these formations were artificially enhanced (see text for the possible origin of these formations). Scale bar, 5 µm.

Following this experimental design, we first examined the localization of the minus end binding Asp protein. Surprisingly, at time 0, Asp-GFP was concentrated at or near the kinetochores of 50% of the PRO-METs (*n* = 200; Figure 8). In normal cells, Asp-GFP never accumulates on the kinetochores and is always enriched at the spindle poles (Figure S9). Thus, our observations suggest that upon MT depolymerization, Asp redistributes in the cytoplasm and associates with the kinetochores. Notably, after 3 h of colcemid treatment, the spindles of more than 95% of PRO-METs are completely depolymerized and do not exhibit clear remnants of the spindle MTs. However, these cells often show multiple irregularly shaped formations that are weakly immunostained by the anti-α-tubulin antibodies. We do not know the nature of these formations; they might be enriched in tubulin-containing components such as small MT fragments, tubulin dimers/monomers, and tubulin-colcemid aggregates. Interestingly, some of these formations lay close to the kinetochores and to the Asp-GFP signals. This observation is difficult to explain and is subject to several interpretations. It might reflect a simultaneous interaction of Asp with both the tubulin-enriched formations and one or more kinetochore proteins. Alternatively, some of the tubulin-enriched formations might be produced by colcemid-induced MT depolymerization and remain associated with both Asp and the kinetochores.

**Figure 9:**
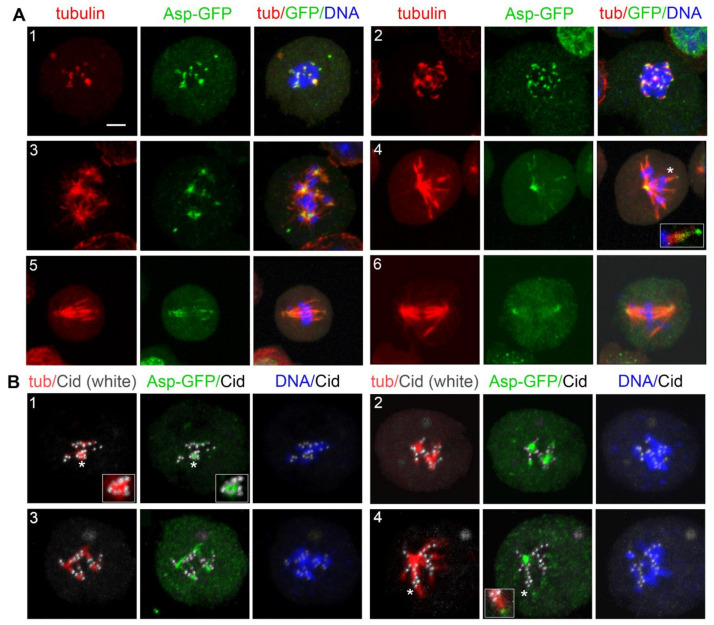
Asp-GFP localization during kinetochore-driven microtubule regrowth (KDMTR) after colcemid-induced MT depolymerization. (**A**) S2 cells expressing both Asp-GFP and Cherry-tubulin were fixed and stained with anti-α-tubulin (red) and anti-GFP (green) antibodies and counterstained for DNA (DAPI, blue). Panel (**A1**) shows an early KDMTR stage; panels (**A2**,**A3**) show more advanced KDMTR phases characterized by the presence of MT clusters/asters. Note that Asp-GFP is localized at the center of these MT structures, suggesting that they correspond to mini spindle poles. Panels (**A4**–**A6**) illustrate one of the modalities of spindle reformation after colcemid-induced MTs depolymerization. Panel (**A4**) shows a representative umbrella-shaped monopolar spindle with Asp-GFP concentrated at the pole. In this regrowing spindle, probably originated by the coalescence of “mini poles”, Asp-GFP also localizes to the extended MT bundles and accumulates at their distal ends (see magnified inset in A4). This localization pattern is likely to reflect the Asp movement along the spindle MTs (see text). Panels (**A5**,**A6**) illustrate how the diverging ends of MT bundles eventually coalesce, giving rise to a bipolar spindle with Asp-GFP enriched at the poles. Scale bar, 5 µm. (**B**) S2 cells expressing both Asp-GFP and Cherry-tubulin were fixed and stained with anti-α-tubulin (red), anti-GFP (green), and anti-Cid (white) antibodies and counterstained for DNA (DAPI, blue). Representative examples of early (**B1**,**B2** and magnified inset in B1) and late (**B3**,**B4** and magnified inset in B4) stages of spindle regrowth after colcemid-induced MT depolymerization illustrating the relationships between Asp-GFP, tubulin, and centromeres (marked by Cid). Note that in all cases, the Asp-GFP signals are surrounded by the centromeres, indicating that the MT clusters/asters are in fact generated by the coalescence of the minus ends of MT bundles emanating from the kinetochores. Scale bar, 5 µm.

At the very early KDMTR stages, Asp-GFP was still accumulated at the kinetochores, showing partial colocalization with the initial tubulin foci. Asp-GFP was then found at the center of the tubulin cluster/asters (Figure 9). With progression of MT regrowth, Asp-GFP associated with the extended MT bundles and accumulated at their minus ends (Figure 9(A4); see also Figure S10). This finding is consistent with previous work showing that in normal spindles, Asp moves along the MTs towards the spindle poles [70,82]. Importantly, when the Asp-GFP signal was located at the center of the tubulin clusters/asters, the Cid (CENPA) centromeric signals were not at the center of these structures but were instead surrounding them (Figure 9B). These results suggest that Asp localizes at the MT minus ends at all regrowth stages, and that the aster-like structures are formed by the coalescence of the Asp-associated minus ends of the MTs emanating from kinetochores. Thus, these MT clusters/asters have features in common with the spindle poles and can be regarded as mini spindle poles.

**Figure 10:**
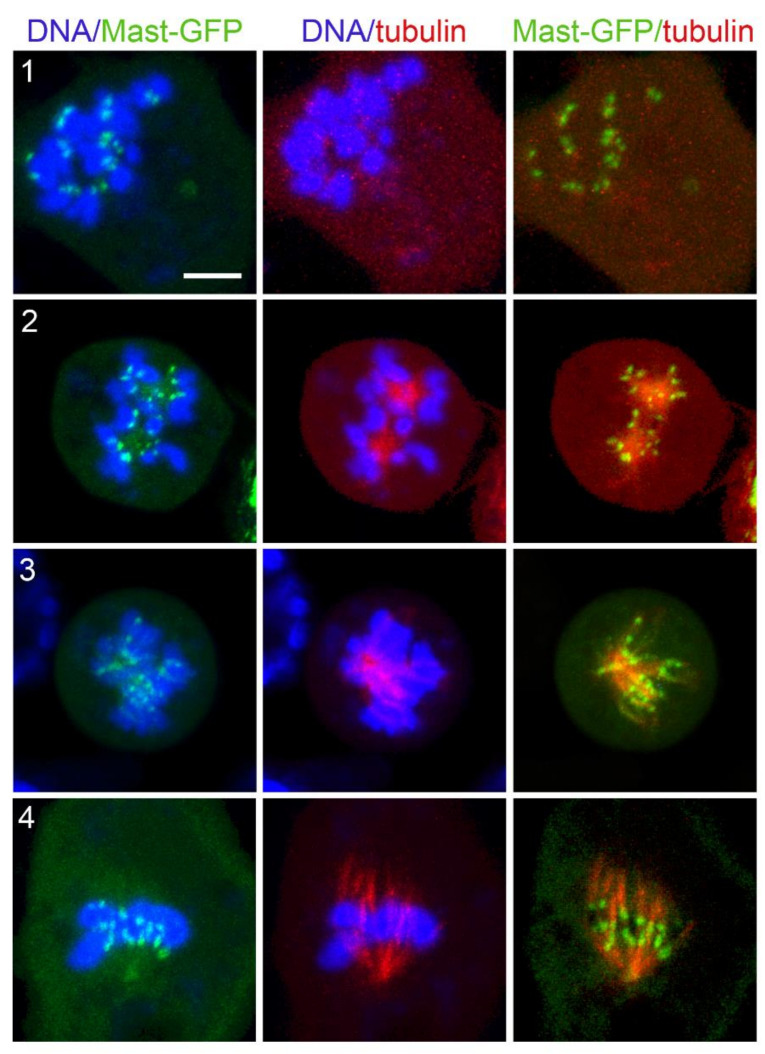
Mast-GFP localization during kinetochore-driven microtubule regrowth (KDMTR) after colcemid-induced MT depolymerization. Live S2 cells expressing Mast-GFP (green) and Cherry-tubulin (red) were stained with the vital DNA stain Hoechst 33342 (blue). Mast-GFP is associated with discrete sites on the chromosomes (probably corresponding to the kinetochores) after 3 h colcemid treatment, before washout of the drug (panel (**1**)). During KDMTR, Mast-GFP is still accumulated on the kinetochores, which surround tubulin clusters/asters (likely mini spindle poles) that exhibit weak GFP fluorescence (panel (**2**)). Mast-GFP remains concentrated on kinetochores during further KDMTR stages (panels (**3**,**4**)). Scale bar, 5 µm.

Similar to Asp, Mast-GFP was accumulated on kinetochores at time 0, when KDMTR was not detectable (Figure 10, panel 1). Thus, Mast-GFP remains associated with kinetochores throughout the process of MT depolymerization. This suggests that Mast (CLASP1) is anchored to the kinetochore by some kinetochore-bound proteins. Although there is no experimental evidence supporting this occurrence in *Drosophila* S2 cells, studies in human cells and *C. elegans* have shown that the CLASP proteins physically interact with CENPE and CENPF orthologs, respectively [83,84]. Mast-GFP accumulation at the kinetochores persisted throughout the process of spindle reassembly, and Mast-GFP signals surrounded the MT clusters/asters (Figure 10). Mast-GFP also associated with both long and extended MT bundles, which, however, showed a much lower GFP fluorescence intensity than the kinetochores (Figure 10).

**Figure 11:**
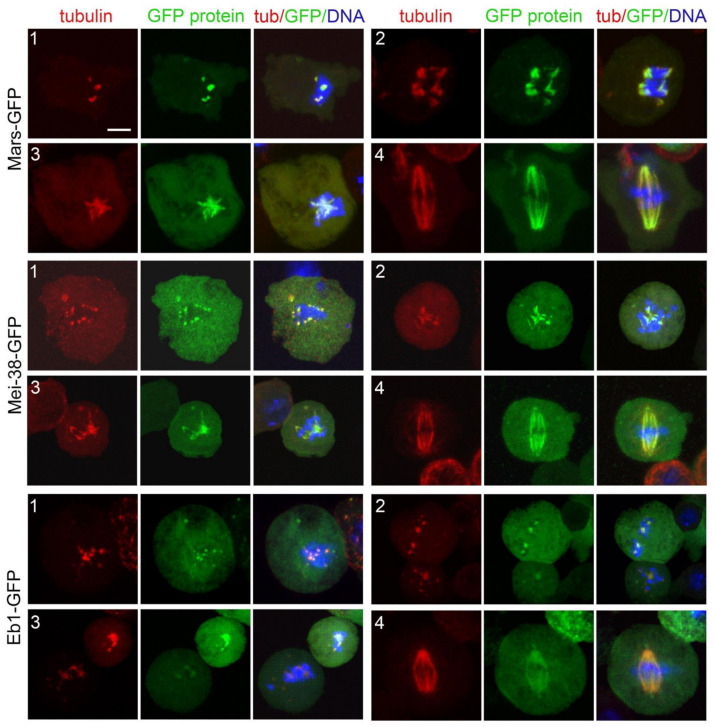
Localization of Mars-GFP, Mei-38-GFP, and Eb1-GFP during kinetochore-driven microtubule regrowth (KDMTR) after colcemid-induced MT depolymerization. Fixed S2 cells expressing the indicated GFP-tagged protein were stained with anti-GFP antibodies (green), anti-α-tubulin antibodies (red), and counterstained for DNA (DAPI, blue). Panel (**1**) shows the initial KDMTR phases with small chromosome-associated tubulin signals that colocalize with the GFP proteins. Panels (**2**,**3**) show more advanced KDMTR phases characterized by the presence of MT clusters/asters. Panel (**4**) shows reformed spindles. Note that in all cases, the tubulin signals colocalize with the GFP signals. After colcemid treatment, the Eb1 comets are small and Eb1 seems to be uniformly distributed along the regrowing MT structures. Scale bar, 5 µm.

When spindle was completely depolymerized (time 0), Mars-GFP, Mei-38-GFP, and Eb1-GFP were not associated with kinetochores. Mars-GFP and Mei-38-GFP colocalized with the initial MT foci at the beginning of spindle regrowth and remained on the spindle until it was completely reformed (Figure 11). Eb1-GFP was also permanently colocalized with the regrowing spindle structures. We filmed Eb1-GFP behavior at early regrowth stages, but we did not see the dynamic MT plus end accumulations (comets) that are typically observed in normal PRO-METs. Regrowing MT structures were instead showing very small Eb1-GFP puncta that appeared to move along short paths both away and towards the kinetochores. However, many completely reformed spindles displayed comets moving from the poles towards the spindle equator, such as in untreated Eb1-GFP expressing cells (Movies S1–S3). These observations are consistent with previous work on acentrosomal spindles assembled in Cnn-depleted cells [16] and suggest that, once formed, the poles of the acentrosomal spindles can drive MT formation.

**Figure 12:**
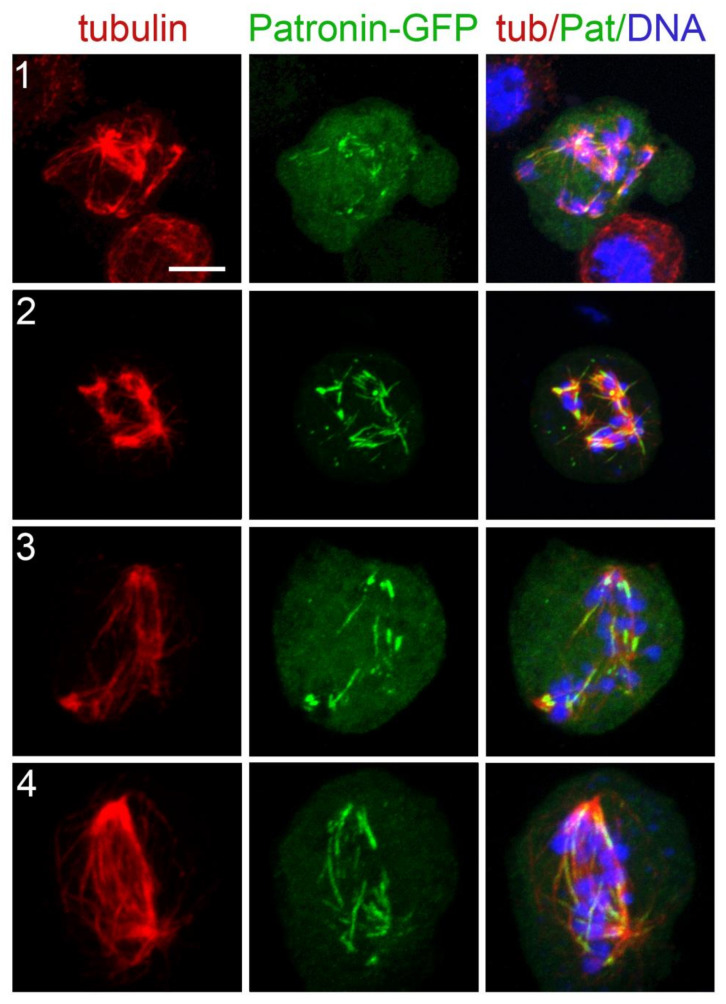
Patronin-GFP localization during kinetochore-driven microtubule regrowth (KDMTR) after colcemid-induced MT depolymerization. S2 cells expressing both Patronin-GFP and Cherry-tubulin were fixed and stained with anti-α-tubulin (red) and anti-GFP (green) antibodies and counterstained for DNA (DAPI, blue). In very early KDMTR stages, the highly fluorescent form of Patronin-GFP is not visible (see text). At later KDMTR stages, characterized by MT clusters/asters formation, bright Patronin-GFP is observed within these structures (panels (**1**,**2**)) where it localizes along some but not all MT bundles. Localization along a fraction of the extended MT bundles is also seen in advanced stages of spindle reformation (panels (**3**,**4**)). Scale bar, 5 µm.

As pointed out earlier, in untreated cells, the fluorescence of spindle-associated Patronin-GFP can either be dull or bright. Specifically, Patronin-GFP is bright when it moves from the kinetochores to the spindle poles along the k-fibers [55]. Here, we considered only the bright fluorescence, as the dull one is difficult to score. PRO-METs at time 0 or at the very early KDMTR stages did not show any bright Patronin-GFP signal. These signals were seen in many MT clusters/asters, but their shapes were very different from those produced by Asp-GFP. They appeared as short bars within the MT clusters and were often associated with the extended MT bundles emanating from the clusters/asters (Figure 12). These observations suggest that the bright form of Patronin-GFP marks the kinetochore-driven MTs when they start forming long bundles and moves along these bundles as they keep growing.

## 4. Discussion

### 4.1. Genetic Control of MT Regrowth after Tubulin Depolymerization

We have shown that the mechanisms underlying KDMTR following colcemid-induced MT depolymerization can be genetically dissected using RNAi. In *mast*, *mei-38*, *mars*, *Eb1*, and *dgt6* RNAi cells, KDMTR is strongly reduced compared to control. In Patronin-depleted cells, the advanced regrowth figures (long MT bundles and MT clusters/asters) are slightly but not significantly less frequent than in control cells, but the mean CATF is significantly lower than in control. This finding could reflect a minor role of Patronin in KDMTR but could also be a consequence of the limited efficiency of RNAi against *Patronin*. In our experimental conditions, *Patronin* RNAi cells showed 28% of residual mRNA, while the residual mRNAs for the other genes ranged from 3 to 8% of the control level (Figure 1). In contrast, depletion of either Klp10A or Asp promotes KDMTR.

Previous work has suggested that in S2 cells lacking functional centrosomes, kinetochore-driven MT formation is not controlled by the RCC1-RanGTP pathway [28]. Nonetheless, we found that the *Drosophila* homologs of TPX2 (Mei-38) and HURP (Mars) are required for KDMTR just as their vertebrate counterparts that are regulated by RanGTP [22,33]. The *Xorbit* gene, the *Xenopus* homolog of *mast* (*CLASP1*), has also been shown to be required for chromatin-induced MTs nucleation [85], but there is no published evidence that the CLASP proteins control kinetochore-dependent MT formation in mammalian cells. To the best of our knowledge, the possible roles of the vertebrate homologs of Eb1, Asp, and Klp10A in KDMTR have never been addressed. However, given that at least Eb1, Asp, and Klp10A appear to play conserved mitotic functions, it is quite possible that their vertebrate orthologues also control KDMTR.

We unexpectedly found that after colcemid- or cold-induced MT depolymerization, control and RNAi cells display very similar KDMTR figures and that the main difference between control and RNAi samples was in the frequency of these figures at different fixation times (Figure 3, Figure 5, and Figure 7). Thus, it appears that our RNAi treatments either delay or stimulate KDMTR without affecting the pattern of MT regrowth. This observation points to a particular genetic robustness of the KDMTR process and suggests that it is under the control of many genes that are at least in part functionally redundant. This is not surprising, given that an efficient cell division is at the basis of life and that the kinetochore-driven MT formation pathway is not only necessary but also sufficient for spindle assembly in most eukaryotic cells.

### 4.2. Kinetochore-Driven MT Growth and Spindle Length

Our results indicate that the efficiency of KDMTR in RNAi cells positively correlates with the length of the spindles observed in the same cells. For example, the spindles of Mast-, Mei-38-, Mars-, Dgt6-, Patronin-, and Eb1-depleted cells are significantly shorter than control spindles, while the spindles of *asp* RNAi cells are longer than in control (Figure S2A; see also [24,55,61,62,63]). Further, the spindles of Klp10A-depleted cells are longer than control spindles; the increased KDMTR and spindle length in these cells are a likely consequence of a reduced Klp10A-mediated MT depolymerization [61,63]. Other *Drosophila* proteins that promote KDMTR and whose loss results in short spindles are Misato (Mst) and its interacting partners of the TCP-1 and Prefoldin complexes [34,35], Ensconsin that shares homology with human MAP7 [36], and the Msps (TOGp) MT polymerase [24,61]. Thus, at least in *Drosophila* S2 cells, KDMTR effectiveness strongly correlates with the length of prometaphase/metaphase spindles. RNAi-based screens in S2 cells identified many additional genes associated with a short spindle phenotype [17,74] but whether their loss results in decreased KDMTR is currently unknown.

### 4.3. Kinetochore-Driven MT Growth during Normal Mitosis and after MT Depolymerization

One of the main questions is whether and to what extent KDMTR recapitulates the MT growth that occurs at kinetochores during normal cell division. As mentioned earlier, centrosome-containing S2 cells were used for a pivotal analysis of kinetochore-driven MT growth. Maiato and coworkers (2004) [21] showed that living cells stably expressing GFP-α-tubulin form k-fibers from the unattached kinetochore of mono-oriented chromosomes and that these fibers extend towards the cell periphery until they interact with the astral MTs and become oriented towards the spindle poles. They also used laser beam microsurgery to show that when individual k-fibers are severed, the fragment associated with the kinetochore (k-fragment) regrows, while the fragment terminating in the spindle pole degenerates. Finally, using an elegant combination of laser microsurgery/photobleaching, they were able to demonstrate that the growth of the k-fragment starts at or near the kinetochore. Based on these data, they proposed that kinetochore-driven MT formation begins with the generation of short, randomly oriented MTs near the kinetochores. The plus ends of these MTs would then be captured by the kinetochore and continue to polymerize there, leading to k-fiber elongation. As a result, the MT minus ends would be pushed away from the kinetochores and accumulate at the end of the regrowing k-fiber [21].

One aspect of this model that has been matter of debate is the mechanism underlying the initial MT regrowth. Mishra and coworkers (2010) [39] found that Nup107-160 and γ-tubulin localize next to the kinetochores in HeLa cells at the early MT regrowth stages after nocodazole-induced MT depolymerization. They also showed that KDMTR is reduced in the absence of either Nup107-160 or γ-tubulin. They concluded that although these results are compatible with the Maiato’s model [21], they are also consistent with the possibility that during the initial stages of KDMTR, MTs are anchored to the kinetochores through their minus ends and polymerize at the distal plus ends, forming an assembly intermediate that disappears as k-fibers elongate [39]. A similar transient polarity inversion of kinetochore MTs has been described in budding yeast, where kinetochores are thought to generate MTs with the minus ends embedded into the kinetochores and the plus ends pointing outwards. However, these MTs do not contribute to metaphase spindle formation, as they disappear when the plus ends of the MTs emanating from the spindle poles are captured by the kinetochores [86].

We have shown that Asp-GFP accumulates at kinetochores when KDMTR is not detectable and then partially colocalizes with the first kinetochore-associated small foci of regrowing MTs. As KDMTR proceeds, Asp-GFP is consistently found at the center of MT clusters/asters regardless of their size. These structures do not exhibit kinetochores at their centers but are instead surrounded by kinetochores. This suggests that Asp-GFP binds the minus ends of the regrowing MT bundles since the beginning of their formation and that the minus ends of these bundles eventually converge giving rise to a sort of mini spindle poles with Asp-GFP at their center. Asp-GFP then localizes on the extended MT bundles/k-fibers that emanate from these clusters/asters and accumulates at their extremities, indicating that they are indeed enriched in MT minus ends [21]. The simplest interpretation of these observations is that Asp-GFP associates with the MT minus ends emanating from kinetochores and is pushed away by the growth of the MT plus ends embedded into these organelles. Thus, our data argue against the possibility of a transient MT polarity inversion during the early stages of MT regrowth after colcemid-induced depolymerization.

Recent ultrastructural studies revealed that most kinetochores of unperturbed human cells in very early prometaphase are transiently associated with a mesh of short randomly oriented noncentrosomal MTs, which is no longer observed upon formation of mature k-fibers [26]. Although there is no direct evidence that this MT mesh is present also in early S2 cell prometaphases, our data are consistent with its existence at least in cells undergoing KDMTR. It is likely that Asp-GFP binds the minus ends of the short MTs that form near the centromeres, limiting their growth, as suggested by the finding that in Asp-depleted cells, KDMTR is increased compared to control. In human cells, ASPM forms a complex with katanin, and the two proteins cooperate in promoting MT severing [68]. Whether katanin binds *Drosophila* Asp is unknown, but if so, katanin might contribute to the regulation of MT minus ends also during S2 cells mitosis. The finding that during KDMTR Asp is transiently associated with the kinetochores further suggests that this protein might have role in the establishment of a correct kinetochore-MT attachment and explains why Asp-depleted cells suffer a spindle checkpoint-dependent metaphase arrest [87].

### 4.4. An Integrated Model for Kinetochore-Driven MT Growth

Our results provide insight into the genetic/molecular control of KDMTR. Previous work on *Drosophila* S2 cells has shown that Mast mediates the incorporation of tubulin subunits into the plus ends of the mature k-fibers embedded into the kinetochore [71]. It has been also suggested that human CLASP1 plays a similar function [88]. Our findings that depletion of Mast results in a strong reduction of KDMTR and that Mast-GFP co-localizes with the kinetochores in all phases of MT regrowth strongly suggest that this protein is the main determinant of MT plus end elongation during KDMTR.

We found that Mars (HURP), Mei-38 (TPX2), and the augmin subunit Dgt6 are required for KDMTR. Studies in vertebrate systems have shown that TPX2 interacts with a variety of mitotic proteins, including Aurora A (AURKA) and the augmin complex; promotes MT nucleation and bundling; and is required for both kinetochore fiber formation and KDMTR [3,22,89]. HURP has MT bundling activity and is required for k-fiber stability and KDMTR [23,90]. Augmin is thought to promote MT nucleation within the k-fibers by recruiting the γ-TURCs (reviewed in [3]). We have shown that these proteins precisely colocalize with the kinetochore-driven MTs from the very early stages of regrowth to the complete reformation of the spindles. This suggests that these three proteins play conserved functions required for proper reassembly and stability of k-fibers after MT depolymerization.

We also found that KDMTR is negatively affected by loss of Eb1 and at least in part by Patronin depletion. The two proteins showed very different patterns of localization during KDMTR. Eb1-GFP localized to the kinetochore-dependent MTs from the beginning of repolymerization and remained on these MTs until the spindle was reassembled. In contrast, the brightly fluorescent form of Patronin-GFP was not observed in early KDMTR stages but localized to long and extended MT bundles within clusters/asters and reforming spindles. Our analyses on unperturbed live and fixed cells showed that brightly fluorescent Patronin-GFP is highly dynamic and tends to move from the kinetochores to the spindle poles in unperturbed S2 cells [55]. We speculate that Patronin-GFP moves along the regrowing MT bundles exploiting an as yet unidentified minus end-directed motor. During its movement, Patronin-GFP would associate with the MT minus ends, including those generated by the augmin pathway. This would help maintaining the correct structural organization and stability of mitotic MT bundles [55]. Notably, the human homologs of Patronin (CAMSAP2, CAMSAP1, CAMSAP3) bind katanin similarly to ASPM [91] but it is currently unknown whether this occurs also in *Drosophila*. In any case, our results suggest that loss of Patronin does not result in reduced severing of spindles MTs.

In summary, our results indicate that KDMTR after MT depolymerization recapitulates the process of kinetochore-driven MT formation in unperturbed cells. Our results also suggest an integration of the current model for kinetochore-dependent MT formation in *Drosophila* S2 cells [21]. We propose that kinetochores capture the plus ends of MTs nucleated in their vicinity and that these MTs elongate through the action of Mast. These processes are likely to be downregulated by Asp that binds the MT minus ends and prevents excessive and disorganized KDMTR. Mars, Mei-38, Dgt6, Eb1, and Patronin positively regulate the subsequent formation, elongation, and stabilization of the regrowing MT bundles/k-fibers.

## Data Availability

Data is contained within the article or Supplementary Materials.

## Supplementary Materials

The following supporting information can be downloaded at: https://www.mdpi.com/article/10.3390/cells11142127/s1, Figure S1: Examples of mitotic cells observed after RNAi against *mast*, *mei-38*, *Eb1*, *Patronin*, *asp*, *mars*, *dgt6*, and *Klp10A*. Figure S2: Quantitation of mitotic phenotypes observed after RNAi against *mast*, *mars*, *mei-38*, *Eb1, dgt6*, *Patronin*, *asp*, and *Klp10A*. Figure S3: A representative example of spindle reformation after colcemid-induced MT depolymerization. Figure S4: Pattern of kinetochore-driven microtubule regrowth (KDMTR) observed in control cells at different times after colcemid washout. Figure S5: Mast-GFP localization during mitosis of S2 cells. Figure S6: Mei-38-GFP localization during mitosis of S2 cells. Figure S7: Mars-GFP localization during mitosis of S2 cells. Figure S8: Eb1-GFP localization during mitosis of S2 cells. Figure S9: Asp-GFP localization during mitosis of S2 cells. Figure S10: A representative example of spindle reformation after colcemid-induced MT depolymerization in S2 cells expressing both Asp-GFP and Cherry-tubulin. Table S1: dsRNAs used for RNA interference. Table S2: Primers used for the assessment of the RNAi-mediated gene silencing by RT-qPCR. Movie S1: Microtubule dynamics in an unperturbed S2 cell prometaphase expressing Eb1-GFP. Movie S2: Microtubule dynamics in a MT cluster during kinetochore-driven microtubule regrowth (KDMTR) after colcemid-induced MT depolymerization in cells expressing Eb1-GFP. Movie S3: Microtubule dynamics in an almost completely reformed prometaphase expressing Eb1-GFP following colcemid-induced MT depolymerization.
