# Supplementary material for: Genetic Control of Kinetochore-Driven Microtubule Growth in Drosophila Mitosis
Source: Cells. 2022 Jul 6;11(14):2127. doi: 10.3390/cells11142127 (PMC9323100; doi:10.3390/cells11142127)
Supplement: Supplementary file 1 [file cells-11-02127-s001.zip › cells-1722269-Figure S1-S10.pdf]

Supporting information

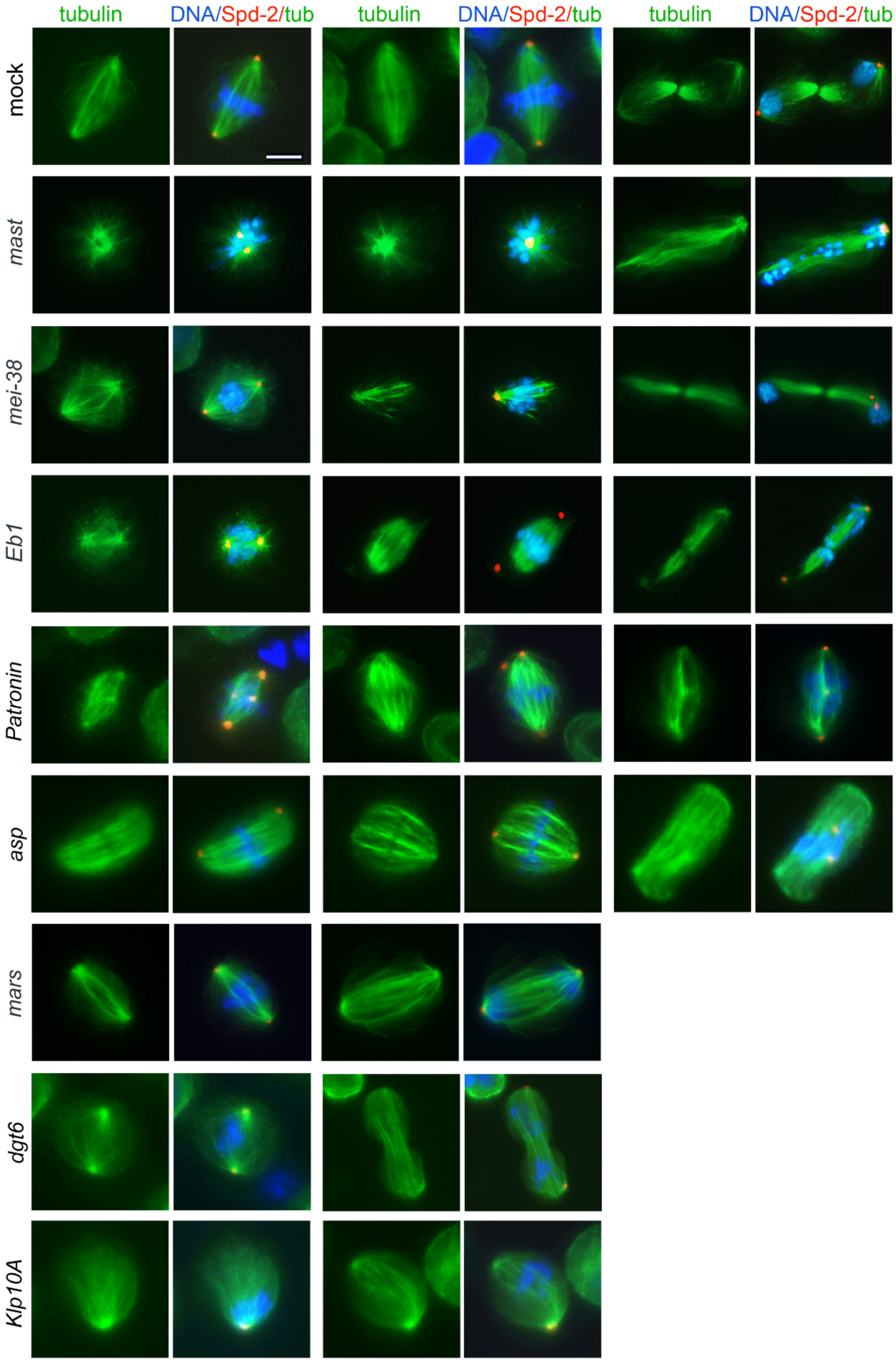

Figure S1. Examples of mitotic cells observed after RNAi against *mast*, *mei-38*, *Eb1*, *Patronin*, *asp*, *mars*, *dgt6* and *Klp10A*. Cells are stained for tubulin (green), the centrosomal marker Spd-2 (red) and counterstained for DNA (DAPI, blue). The following mitotic figures are shown in sequence. Mock: prometaphase, metaphase, telophase; *mast* RNAi: prometaphase with a very short

bipolar spindle, prometaphase with very short monopolar spindle, prometaphase-like cell with elongated spindle (PMLES); *mei-38* RNAi: prometaphase with a bipolar spindle, prometaphase with a monopolar spindle, telophase with two centrosomes at one of the spindle poles and no centrosome at the other; *Eb1* RNAi: prometaphase with a short bipolar spindle, metaphase with both centrosomes detached from the spindle poles, PMLES; *Patronin* RNAi: prometaphase with 4 centrosomes, prometaphase with 3 centrosomes, PMLES; *asp* RNAi: prometaphase with broad spindle poles, metaphase with broad spindle poles, prometaphase with broad poles and the centrosomes detached from the spindle poles; *mars* RNAi: prometaphase and anaphase with short but relatively normal spindles; *dgt6* RNAi: prometaphase and anaphase with short spindles showing a low MT density; *Klp10A* RNAi: monopolar prometaphase; bipolar prometaphase with two centrosomes at one of the spindle poles and no centrosome at other. Scale bar, 5 µm.

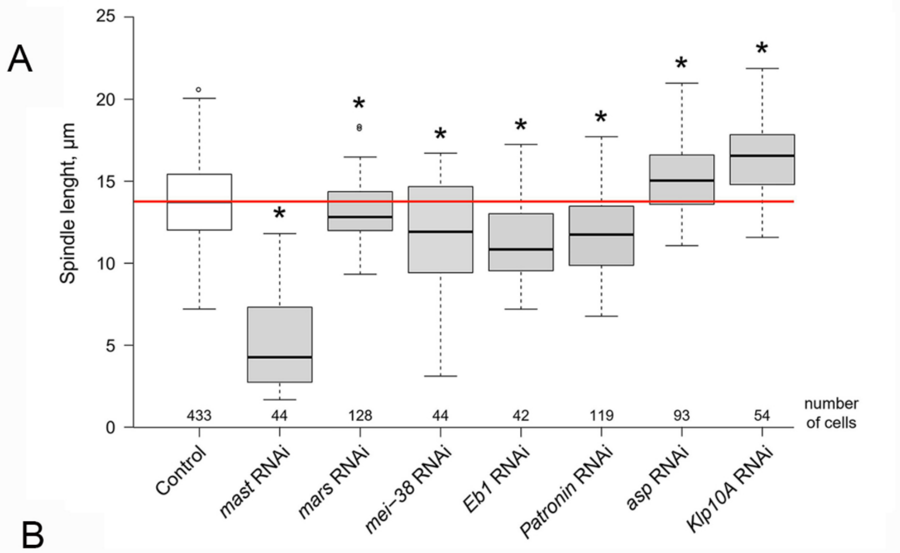

B

Targeted gene	Centrosomes/spindle poles			Distribution of cells by stages of mitosis (%)			
	# of cells scored (1)	Monopolar %	>2 centros. %	# of cells scored (2)	Prometa and Meta	Ana and Telo	PMLES
None (control)	616	3.3	7.6	549	59.4	35.0	5.6
mast	425	49.6*	8.0	181	84.5*	7.7*	7.8
mars	338	6.8§	7.4	290	67.2§	24.5*	8.3
mei-38	458	23.8*	15.7*	277	70.1*	21.2*	8.7
Eb1	356	3.1	10.4	308	62.9	25.8*	11.3*
dgt6	340	14.4*	4.7	275	74.8*	8.2*	17.0*
Patronin	261	6.9§	15.7*	202	58.6	34.8	6.6
asp	262	4.2	5.0	238	69.6*	19.0*	11.4*
Klp10A	356	16.9*	9.8	261	62.7	27.7§	9.6§

Figure S2. Quantitation of mitotic phenotypes observed after RNAi against *mast*, *mars*, *mei-38*, *Eb1*, *dgt6*, *Patronin*, *asp* and *Klp10A*. A. Spindle length in RNAi cells. Consistent with previous results, the spindles of *mast*, *mars*, *mei-38*, *Eb1* and *Patronin* RNAi cells are significantly shorter than control spindles, while those of *asp* and *Klp10A* RNAi cells are significantly longer. The red line represents the median of the control sample. * $p < 0.05$; Mann–Whitney *U* test. **B**. Frequencies of mitotic figures observed in RNAi and control cells. The frequencies of cells with monopolar

spindles and multiple (> 2) centrosomes were determined by examining all mitotic cells (# of cells scored, 1). We did not try to distinguish between monopolar spindles with a single centrosome and monopolars with 2 centrosomes at center of a monaster. The frequencies of the different mitotic figures were determined by examining only bipolar spindles with two centrosomes (# of cells scored, 2). Prometa, prometaphases; Meta, metaphases; Ana, anaphases; Telo, telophases; PMLES, prometaphase-like cells with elongated spindles. § and *, significant in χ^2 with $p \leq 0.05$, and ≤ 0.01,respectively.

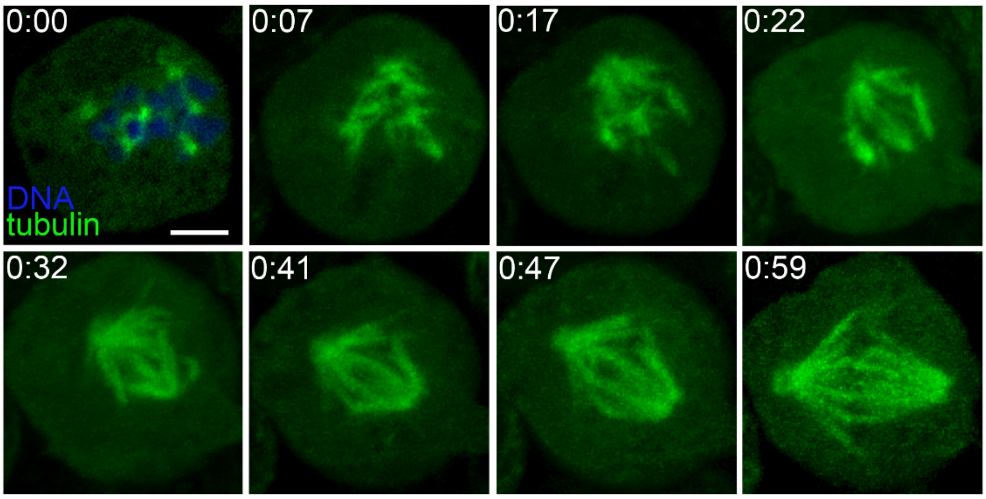

Figure S3. A representative example of spindle reformation after colcemid-induced MT depolymerization. The live S2 cell shown, which expresses GFP-tubulin, was stained with the vital DNA stain (Hoechst 33342 (blue); shown only at time 0:00). Timescale, h:min. Note that at the beginning of imaging (~40 min after colcemid washout; time 0:00) there are only a few small tubulin regrowth foci associated with the chromosomes. These foci grow rapidly forming MT clusters/asters (time 0:07) that coalesce producing a spindle that is focused at only one of the poles (time 0:22). The MT bundles at the unfocused pole progressively converge forming a second pole (time 0:32-0:59). Scale bar, 5 µm.

A

Fixation time	# of cells scored	Short MT bundles per cell	Long MT bundles per cell	Clusters/ asters per cell	Cells with reformed spindles (%)
20 min	85	2.93	0.65	0.02	0.00
30 min	693	3.49	1.74	0.52	0.00
45 min	131	2.54	2.82	1.43	0.00
75 min	143	0.05	0.41	2.25	55.9

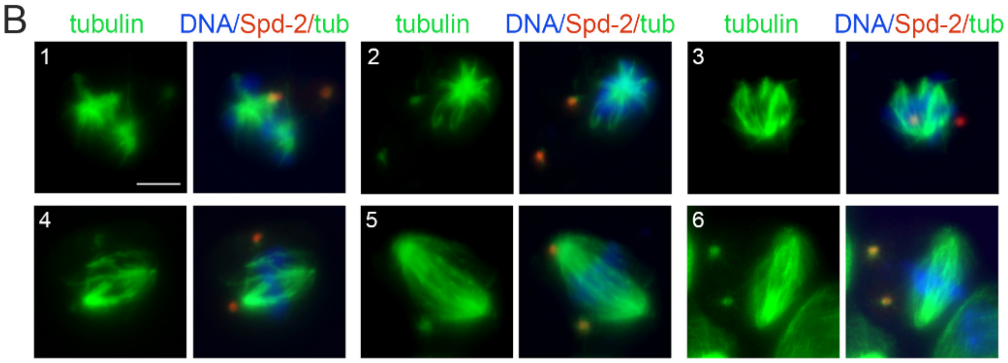

Figure S4. Pattern of kinetochore-driven MT regrowth (KDMTR) observed in control cells at different times after colcemid washout. **A**. Mean numbers per cell of the indicated KDMTR figures observed at different fixation times after colcemid washout. At 75 min fixation time, ~60% of prometaphases and metaphases (PRO-METs) exhibit partially or completely reformed spindles, which are not observed at the earlier fixation times. At 75 min time, the mean numbers of KDMTR figures refer only to the cells that do not show a spindle-like structure. **B**. Examples of MT clusters/asters and partially or completely reformed spindles observed at 75 min fixation time. Cells are stained for tubulin (green), the centrosomal marker Spd-2 (red) and counterstained for DNA (DAPI, blue). Panels 1 and 2 show large chromosome-associated MT clusters/asters. Panels 3 and 4 show incompletely reformed spindles; one of the poles of the spindle in panel 3 is splayed and appears to consist of three polarized MT aggregates. Fully formed spindles are shown in panels 5 and 6. Note that in both incompletely and completely reformed spindle one or both centrosomes are not associated with the spindle poles. This finding indicates that after colcemid-induced MT depolymerization KDMTR can produce well-polarized spindles in the absence of centrosome function. Scale bar, 5 µm.

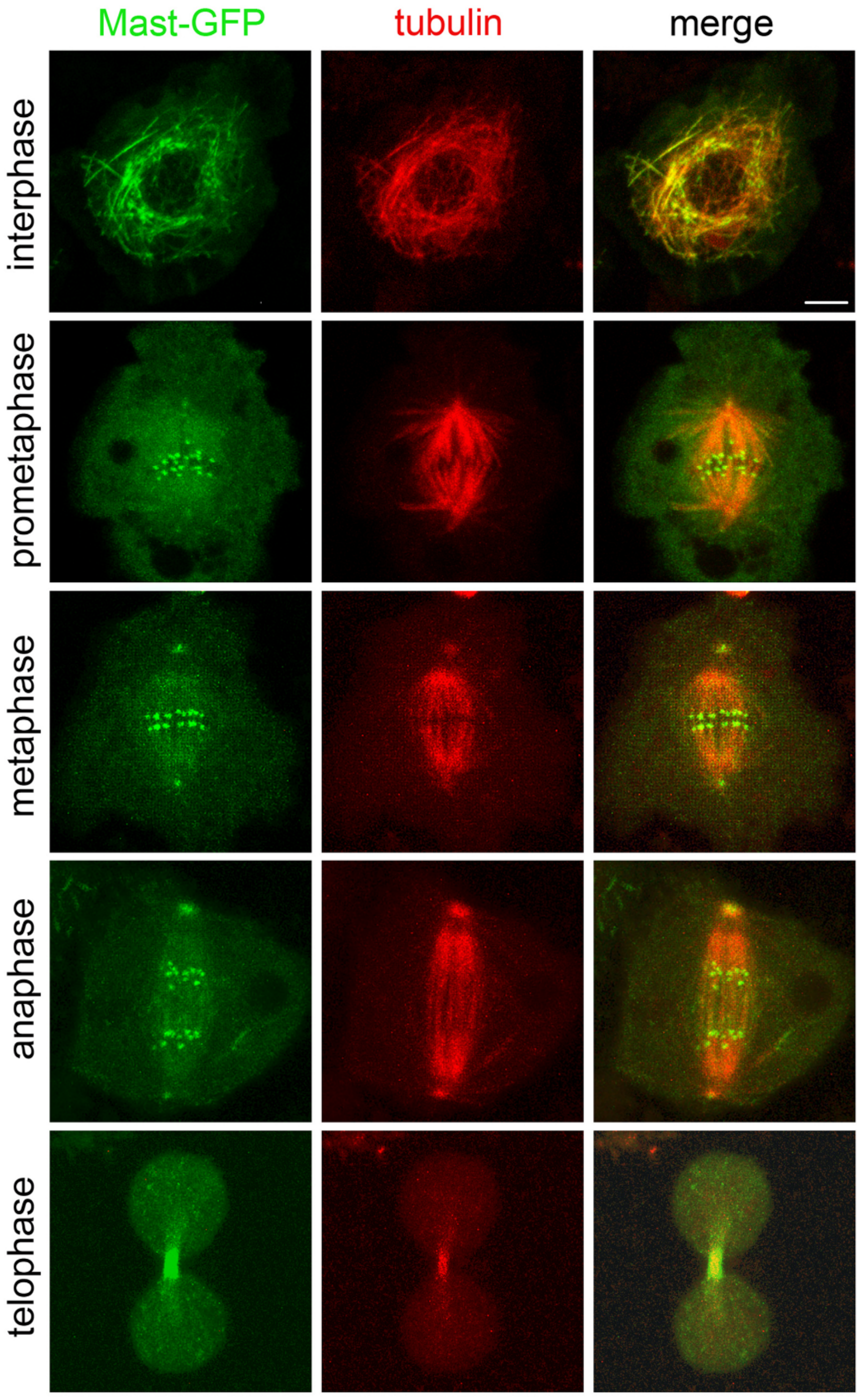

Figure S5. Mast-GFP localization during mitosis of S2 cells. Live interphase and mitotic S2 cells expressing both Mast-GFP and Cherry-tubulin. Note that Mast-GFP accumulates at the kinetochores, the centrosomes and the central spindle of telophase cells. Scale bar, 5 µm.

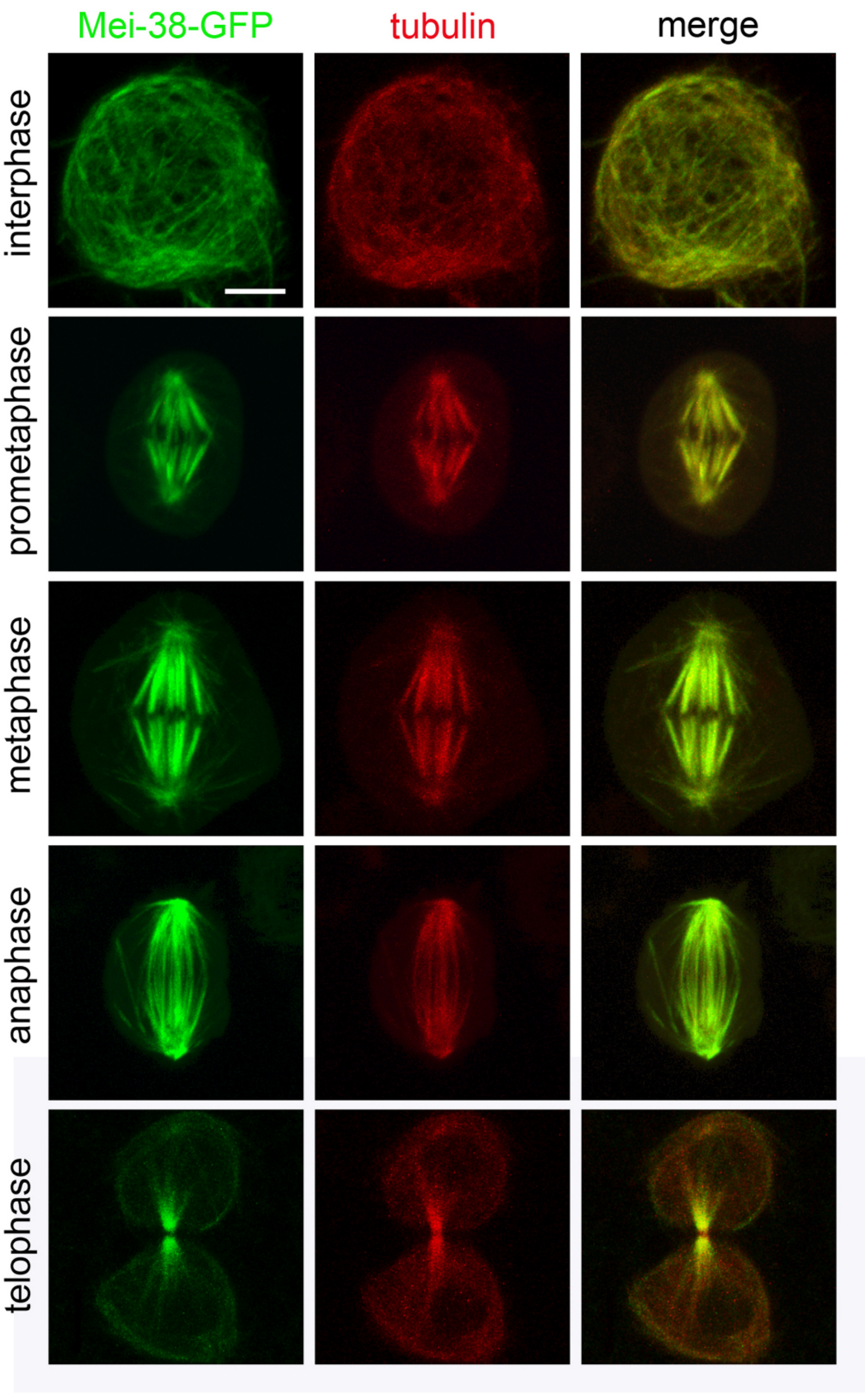

Figure S6. Mei-38-GFP localization during mitosis of S2 cells. Live interphase and mitotic S2 cells expressing both Mei-38-GFP and Cherry-tubulin. Note that Mei-38-GFP associates with the spindle MTs throughout mitosis. Scale bar, 5 µm.

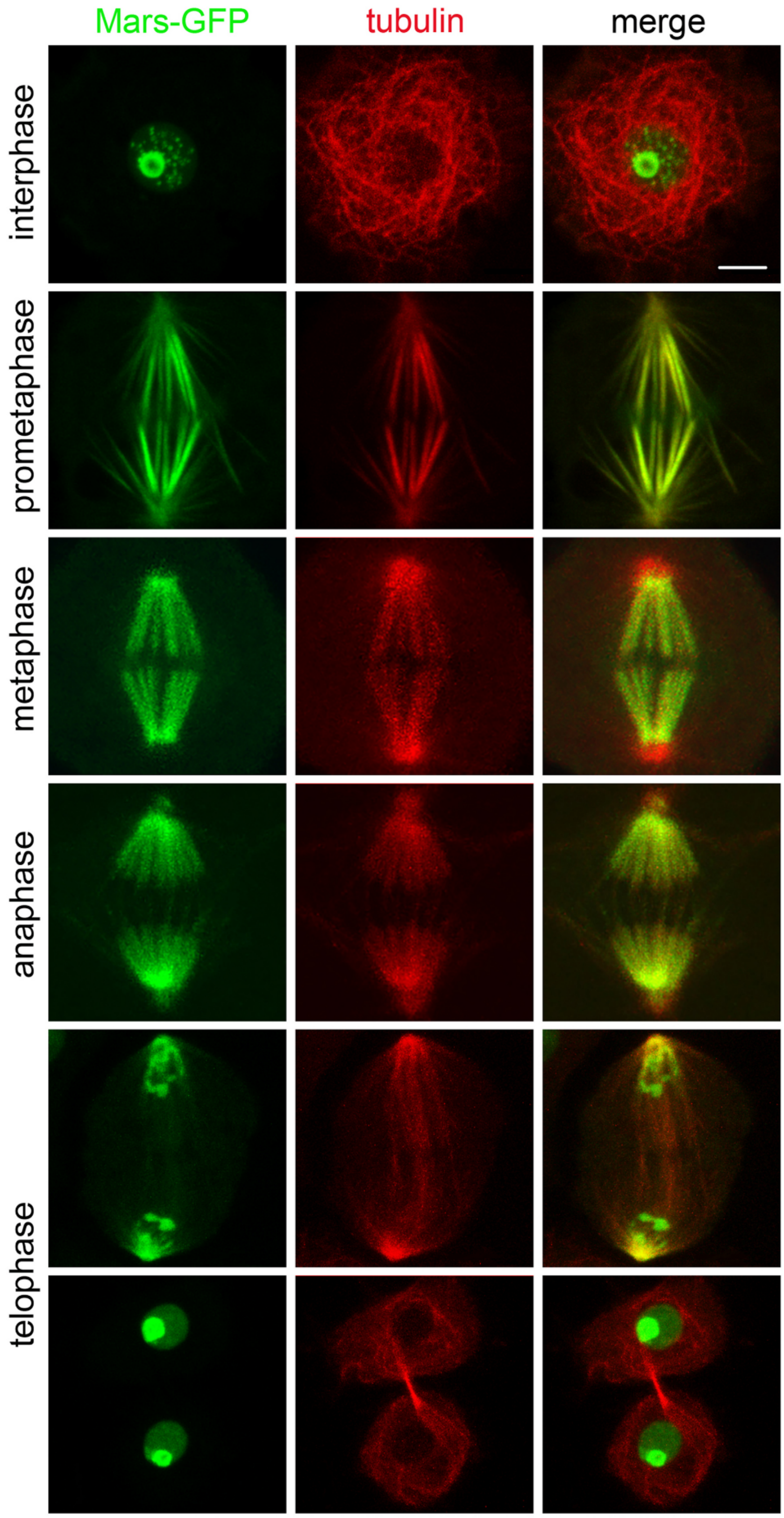

Figure S7. Mars-GFP localization during mitosis of S2 cells. Live interphase and mitotic S2 cells expressing both Mars-GFP and Cherry-tubulin. Note that Mars-GFP accumulates in the nucleolus in interphase and late telophase cells. During metaphase and early anaphase, Mars-GFP associates with the spindle MTs but is excluded from the centrosome area. In late anaphase and telophase, Mars-GFP is no longer associated with the spindle MTs. Scale bar, 5 µm.

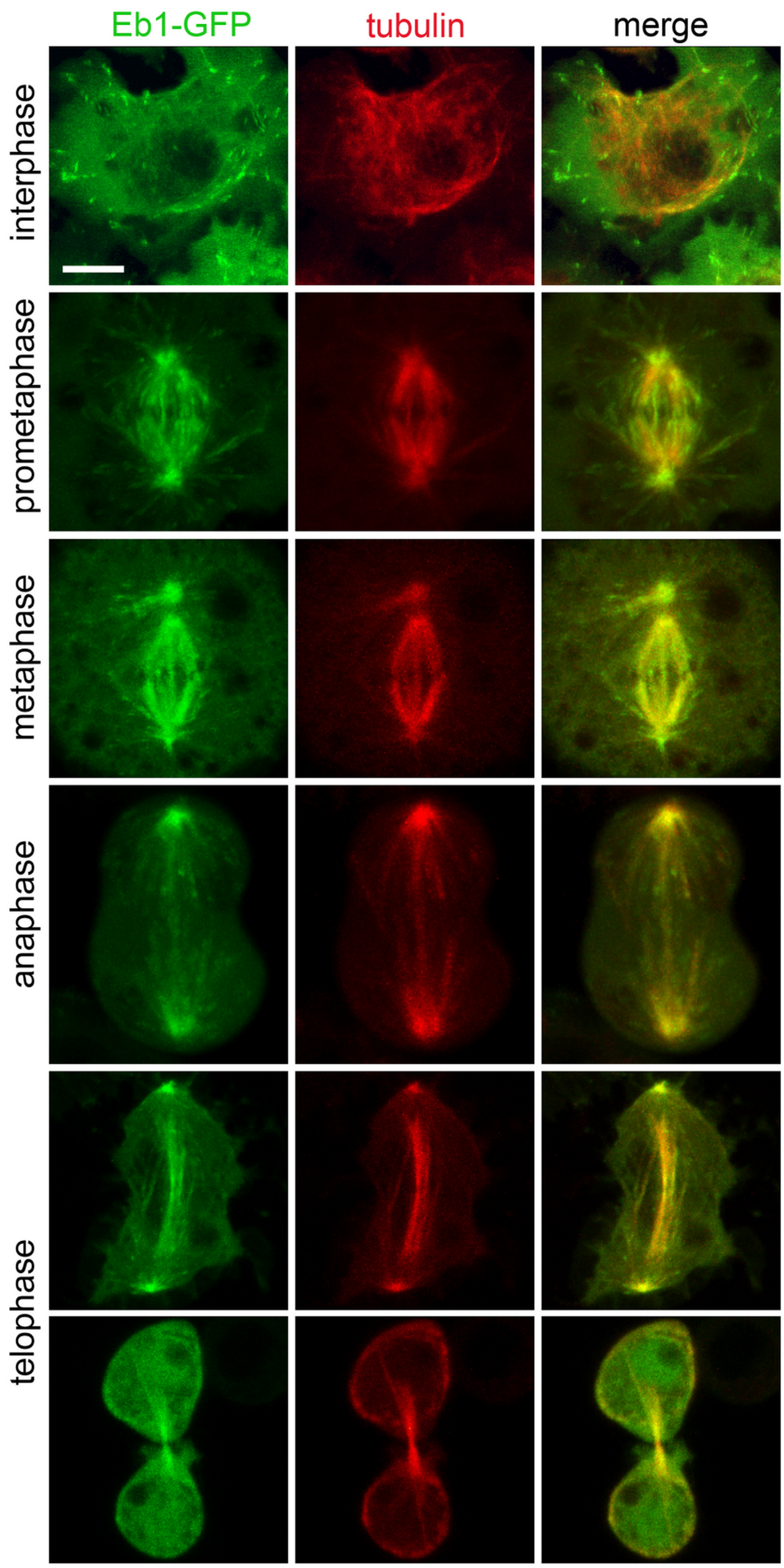

Figure S8. Eb1-GFP localization during mitosis of S2 cells. Live interphase and mitotic S2 cells expressing both Eb1-GFP and Cherry-tubulin. Note that Eb1-GFP associates with the spindle throughout mitosis showing accumulation at the MT plus ends. Scale bar, 5 μm.

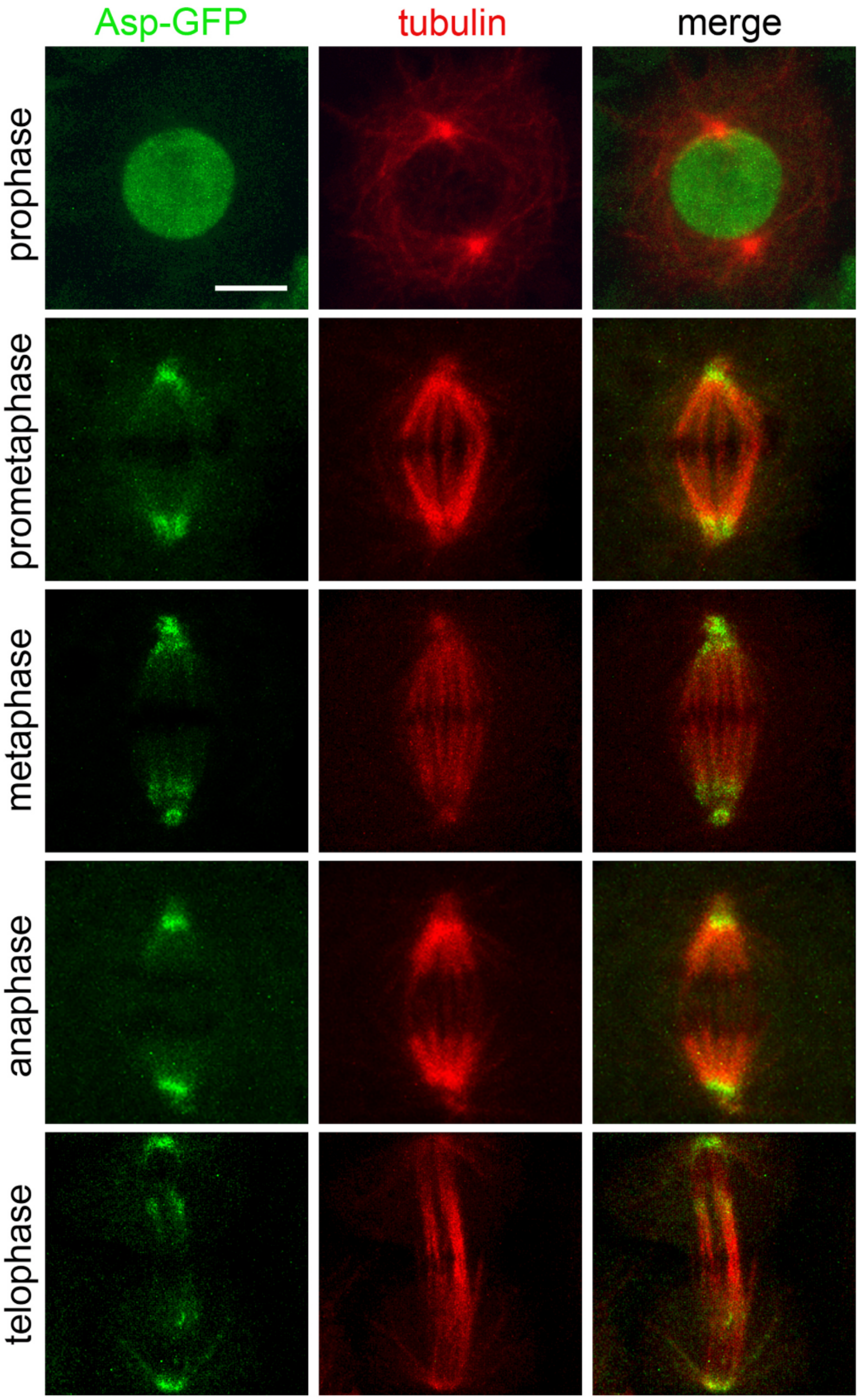

Figure S9. Asp-GFP localization during mitosis of S2 cells. Live interphase and mitotic S2 cells expressing both Asp-GFP and Cherry-tubulin. Note the Asp-GFP accumulation in the interphase nucleus, at the spindle poles and at the MT minus end-enriched extremities of the telophase central spindle. Scale bar, 5 µm.

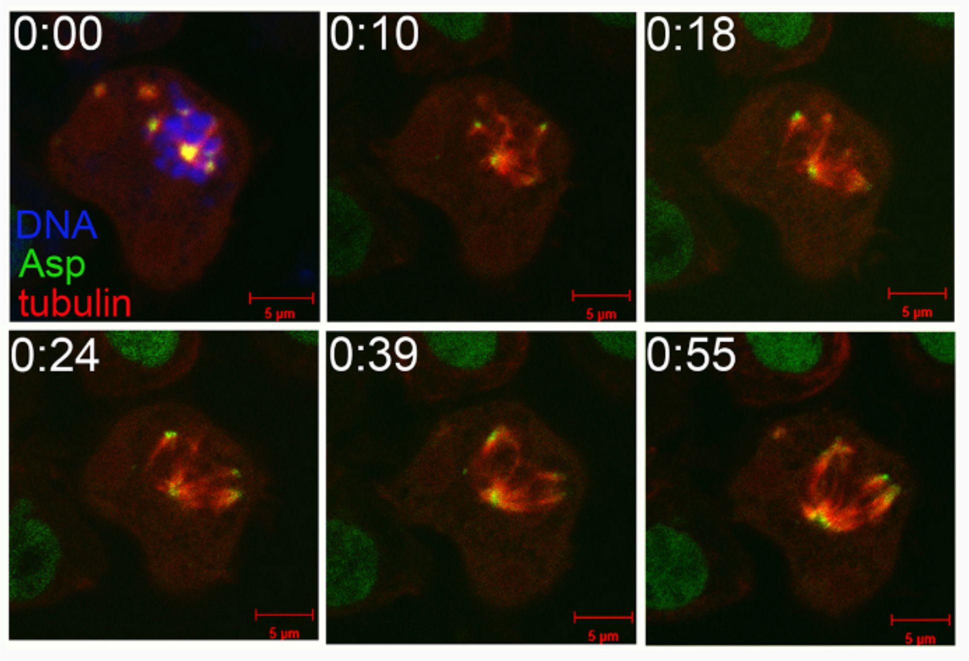

Figure S10. A representative example of spindle reformation after colcemid-induced MT depolymerization in S2 cells expressing both Asp-GFP and Cherry-tubulin. The live S2 cell shown, which expresses both Asp-GFP (green) and Cherry-tubulin (red), was stained with the vital DNA stain (Hoechst 33342 (blue); shown only at time 0:00). Timescale, h:min. Note that at the beginning of imaging (time 0:00) Asp-GFP localizes at the center of MT clusters/asters. Asp-GFP remains in its initial location that defines one of the spindle poles but also moves to the ends of the regrowing MT bundles to form a second spindle pole.

Movie S1. Microtubule dynamics in an unperturbed S2 cell prometaphase expressing Eb1-GFP. The cell is stained with the DNA vital dye Hoechst 33342.

Movie S2. Microtubule dynamics in a MT cluster during kinetochore-driven MT regrowth (KDMTR) after colcemid-induced MT depolymerization. The S2 cell expressing Eb1-GFP is stained with the DNA vital dye Hoechst 33342.

Movie S3. Microtubule dynamics in an almost completely reformed prometaphase spindle following colcemid-induced MT depolymerization. The S2 cell expressing Eb1-GFP is stained with DNA vital dye Hoechst 33342.
