# Supplementary material for: Genetic Control of Kinetochore-Driven Microtubule Growth in Drosophila Mitosis
Source: Cells. 2022 Jul 6;11(14):2127. doi: 10.3390/cells11142127 (PMC9323100; doi:10.3390/cells11142127)
Supplement: Supplementary file 1 [file cells-11-02127-s001.zip › Table S1.pdf]

Table S1. dsRNAs used for RNA interference.

dsRNA probe			Sequences of primers (5′->3′) used to amplify DNA templates for dsRNA synthesis (*)
**Target gene**	**Length, bp**	**Reference**	
asp	959	[1]	CTGCGATCTTTCTTCAG<br>AGATGATTACGCCAATGC
dgt6	785	[2]	ATCGGACCATAA<br>TTGTTCTCGGCT
Eb1	322	This study	TGCTGCCGCGCA<br>TGGTGCATCGTCAGGC
Klp10A	618	This study	TTGCTGTCCATC<br>CGATCCTTGTCT
mars	576	[2]	TGAACTCGCGCT<br>AACTGCTGCAGA
mast	913	This study	CGTTCTCGGAGC<br>ATGTCCACGAAG
mei-38	614	[2]	CGTCCAAGGATG<br>TGGAAGGGTCTG
Patronin	923	[1]	CGAGCTACAGCACCTGTTTC<br>TGACTGATTGCTGACATCGTCC

(*) Each primer contained the following additional sequence of the T7 RNA polymerase-binding site at the 5′ end (5′->3′): TAATACGACTCACTATAGGGAGG.

[1] Pavlova, G.A.; Razuvaeva, A.V.; Popova, J.V.; Andreyeva, E.N.; Yarinich, L.A.; Lebedev, M.O.; Pellacani, C.; Bonaccorsi, S.; Somma, M.P.; Gatti, M.; et al. The role of Patronin in *Drosophila* mitosis. BMC Mol. Cell. Biol. 2019, 20, 7. doi:10.1186/s12860-019-0189-0.

[2] Somma, M.P.; Ceprani, F.; Bucciarelli, E.; Naim, V.; De Arcangelis, V.; Piergentili, R.; Palena, A.; Ciapponi, L.; Giansanti, M.G.; Pellacani, C.; et al. Identification of *Drosophila* mitotic genes by combining co-expression analysis and RNA interference. PLoS Genet. 2008, 4, e1000126. doi:10.1371/journal.pgen.1000126.
