# Supplementary material for: Genetic Control of Kinetochore-Driven Microtubule Growth in Drosophila Mitosis
Source: Cells. 2022 Jul 6;11(14):2127. doi: 10.3390/cells11142127 (PMC9323100; doi:10.3390/cells11142127)
Supplement: Supplementary file 1 [file cells-11-02127-s001.zip › Table S2.pdf]

Table S2. Primers used for assessment of the RNAi-mediated gene silencing by RT-qPCR.

Target gene	Primer sequence (5′->3′)	Primers efficiency, %	Amplicon size (from cDNA), bp
asp	AAGTCGATTGGATCGTCTTTC<br>AATTTAGGATGATCCGGCTG	105.1	155
dgt6	AACAGCTTACTCGCACCTGC<br>GCATGGGATCGTTGATCTTG	101.3	118
Eb1	TCTGCACAGGTGCAGCTTACTGTCA<br>CTTCTTGAAGCCCGCCTGCA	102.9	140
Klp10A	GCTGAGCGAACACGAGATGT<br>CAGTGTGGCATTAACGGTGC	100.2	115
mars	CAGACGACGGTTAAAGAAGACA<br>GCGAGACACTAACAAACGGA	105.9	121
mast	AGACGCTGAGAAATAAACTAGATGC<br>GCAGCTTTGGTGCATGTGTA	106.6	147
mei-38	TTTGGCCAGTCGGACTTCGA<br>CTGTTCCCGGCCGATCTTCT	101.7	165, 168
Patronin	TTTTCAAATACAACTCAGGAGGCA<br>ATTGTGAAGGCGTCGATGGT	99.0	82
RpL32<br>(as reference)	CTAAGCTGTCGCACAAATGG<br>AGGAACTTCTTGAATCCGGTG	100.0	148
